# GLP-1 metabolite GLP-1(9–36) is a systemic inhibitor of mouse and human pancreatic islet glucagon secretion

**DOI:** 10.1007/s00125-023-06060-w

**Published:** 2023-12-21

**Authors:** Nikhil R. Gandasi, Rui Gao, Lakshmi Kothegala, Abigail Pearce, Cristiano Santos, Samuel Acreman, Davide Basco, Anna Benrick, Margarita V. Chibalina, Anne Clark, Claudia Guida, Matthew Harris, Paul R. V. Johnson, Jakob G. Knudsen, Jinfang Ma, Caroline Miranda, Makoto Shigeto, Andrei I. Tarasov, Ho Yan Yeung, Bernard Thorens, Ingrid W. Asterholm, Quan Zhang, Reshma Ramracheya, Graham Ladds, Patrik Rorsman

**Affiliations:** 1 Metabolic Physiology Unit, Department of Physiology, Institute of Neuroscience and Physiology, Sahlgrenska Academy, Gothenburg, Sweden; 2 Cell Metabolism Lab (GA-08), Department of Developmental Biology and Genetics, Indian Institute of Science, Bangalore, India; 3 Oxford Centre for Diabetes, Endocrinology and Metabolism, University of Oxford, Churchill Hospital, Oxford, UK; 4 Department of Pharmacology, University of Cambridge, Cambridge, UK; 5 Center for Integrative Genomics, University of Lausanne, Lausanne, Switzerland; 6 Nuffield Department of Surgical Sciences, John Radcliffe Hospital, Oxford, UK; 7 Biomedical Research Centre, Oxford National Institute for Health Research, Churchill Hospital, Oxford, UK; 8 Section for Cell Biology and Physiology, Department of Biology, University of Copenhagen, Copenhagen, Denmark; 9 School of Biomedical Sciences, University of Ulster, Coleraine, Northern Ireland UK

**Keywords:** GLP-1, *Glp1r*, Glucagon, Glucagon receptor antagonist, Granule docking, Pancreatic alpha cell, Type 2 diabetes

## Abstract

**Aims/hypothesis:**

Diabetes mellitus is associated with impaired insulin secretion, often aggravated by oversecretion of glucagon. Therapeutic interventions should ideally correct both defects. Glucagon-like peptide 1 (GLP-1) has this capability but exactly how it exerts its glucagonostatic effect remains obscure. Following its release GLP-1 is rapidly degraded from GLP-1(7–36) to GLP-1(9–36). We hypothesised that the metabolite GLP-1(9–36) (previously believed to be biologically inactive) exerts a direct inhibitory effect on glucagon secretion and that this mechanism becomes impaired in diabetes.

**Methods:**

We used a combination of glucagon secretion measurements in mouse and human islets (including islets from donors with type 2 diabetes), total internal reflection fluorescence microscopy imaging of secretory granule dynamics, recordings of cytoplasmic Ca^2+^ and measurements of protein kinase A activity, immunocytochemistry, in vivo physiology and GTP-binding protein dissociation studies to explore how GLP-1 exerts its inhibitory effect on glucagon secretion and the role of the metabolite GLP-1(9–36).

**Results:**

GLP-1(7–36) inhibited glucagon secretion in isolated islets with an IC_50_ of 2.5 pmol/l. The effect was particularly strong at low glucose concentrations. The degradation product GLP-1(9–36) shared this capacity. GLP-1(9–36) retained its glucagonostatic effects after genetic/pharmacological inactivation of the GLP-1 receptor. GLP-1(9–36) also potently inhibited glucagon secretion evoked by β-adrenergic stimulation, amino acids and membrane depolarisation. In islet alpha cells, GLP-1(9–36) led to inhibition of Ca^2+^ entry via voltage-gated Ca^2+^ channels sensitive to ω-agatoxin, with consequential pertussis-toxin-sensitive depletion of the docked pool of secretory granules, effects that were prevented by the glucagon receptor antagonists REMD2.59 and L-168049. The capacity of GLP-1(9–36) to inhibit glucagon secretion and reduce the number of docked granules was lost in alpha cells from human donors with type 2 diabetes. In vivo, high exogenous concentrations of GLP-1(9–36) (>100 pmol/l) resulted in a small (30%) lowering of circulating glucagon during insulin-induced hypoglycaemia. This effect was abolished by REMD2.59, which promptly increased circulating glucagon by >225% (adjusted for the change in plasma glucose) without affecting pancreatic glucagon content.

**Conclusions/interpretation:**

We conclude that the GLP-1 metabolite GLP-1(9–36) is a systemic inhibitor of glucagon secretion. We propose that the increase in circulating glucagon observed following genetic/pharmacological inactivation of glucagon signalling in mice and in people with type 2 diabetes reflects the removal of GLP-1(9–36)’s glucagonostatic action.

**Graphical Abstract:**

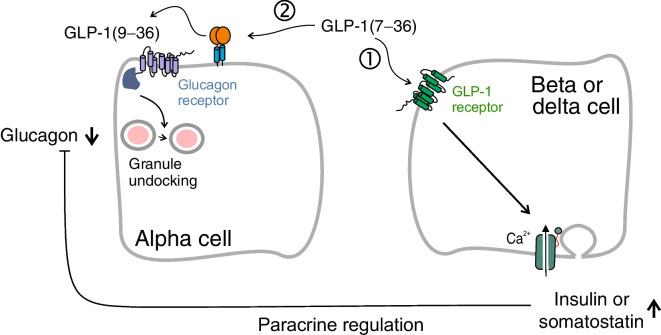

**Supplementary Information:**

The online version of this article  (10.1007/s00125-023-06060-w) contains peer-reviewed but unedited supplementary material.


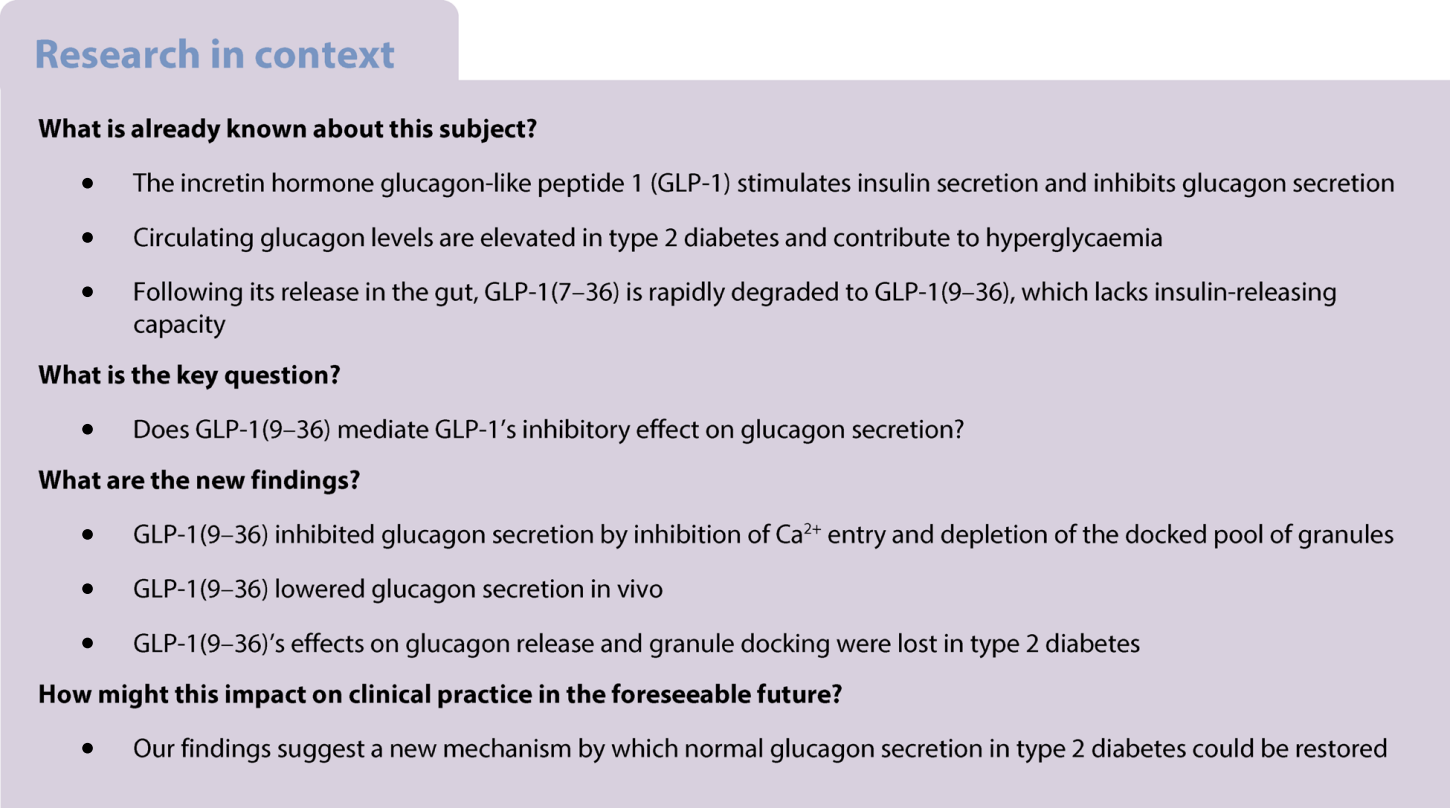


## Introduction

Glucagon is one of the body’s principal blood glucose-increasing (hyperglycaemic) hormones [1]. In type 2 diabetes, the elevation of plasma glucose results from a combination of insufficient insulin and excessive glucagon [2]. Whereas the insulin secretion defect in beta cells has attracted much attention [3], the dysregulation of glucagon secretion in alpha cells represents, by comparison, an understudied area [4].

The incretin hormone glucagon-like peptide 1 (GLP-1) is secreted by the enteroendocrine L cells as GLP-1(7–36). It exerts a strong hypoglycaemic effect by potentiating insulin secretion in beta cells [5] and inhibiting glucagon secretion in alpha cells [6]. Although the latter effect may account for as much as 50% of the peptide’s hypoglycaemic action [6], the underlying cellular mechanism(s) remain(s) poorly understood.

Following its release, GLP-1(7–36) is quickly degraded by dipeptidyl peptidase 4 (DPP-4) to form the metabolite GLP-1(9–36), which lacks insulin-releasing capacity [7], and only 10–15% of the GLP-1 released in the gut reaches the pancreatic islets [5]. We hypothesised that the degradation product GLP-1(9–36) mediates GLP-1’s glucagonostatic effect. Previous in vivo work performed under normoglycaemic conditions has failed to document any effects (stimulatory or inhibitory) of GLP-1(9–36) under normoglycaemic conditions [7–9]. Here, we have compared the glucagonostatic effects of GLP-1(7–36) and (9–36) in vitro using physiological (pmol/l) concentrations of the peptides and over a wider range of glucose concentrations in isolated mouse and human islets (some from donors with type 2 diabetes) and extend these data to the in vivo situation with a particular focus on the counterregulatory increase in plasma glucagon during insulin-induced hypoglycaemia.

## Methods

### Animals and islet isolation

Most studies were conducted in 8- to 16-week-old both male and female C57BL6/J (Envigo, IN, USA) or C57Bl6/N mice (Charles River, MA, USA), which were fed chow (Global Diet no. 2016, Harlan-Teklad). No differences in the secretory function of the two strains were observed. In addition, *Glp1r*^−/−^ mice [10] were used (sex- and age-matched wild-type littermates were used as controls). A transgenic reporter mouse model that expresses a genetically encoded Ca^2+^ indicator GCaMP6-fast variant [11] (GCaMP6f) specifically in alpha cells (Gcg-GCaMP6f) was generated by crossing *Gcg*^CreERT2^ mice [12] (42277-JAX; Jackson Laboratory) (which express a tamoxifen-inducible form of Cre from the endogenous pre-proglucagon gene) to the *Rosa26*^GCaMP6f^ mice [13] (028865; Jackson Laboratory). To induce nuclear accumulation of Cre recombinase and GCaMP6f expression in alpha cells, Gcg-GCaMP6f mice were fed daily with tamoxifen (T5648; Sigma; 20 mg/ml in corn oil) through oral gavage for 5 days. Islets from Gcg-GCaMP6f mice were typically isolated 10 days after tamoxifen induction. The mice were killed by cervical dislocation, the pancreas quickly resected and pancreatic islets isolated by liberase (Sigma) digestion. All experiments in mice were conducted in accordance with the UK Animals (Scientific Procedures) Act (1986), the Ethical Committee at the University of Göteborg or the Veterinary Office of Canton de Vaud.

### Human islets

Human pancreatic islets were isolated (with ethical approval and clinical consent) as previously described [14]. Islets from the pancreases of 18 healthy donors and five donors with type 2 diabetes were used (see electronic supplementary material [ESM] Table 1; ESM Human islets checklist).

### Reagents

GLP-1(7–36), GLP-1(9–36), exendin-4 and exendin(9–39) were from Bachem (Weil am Rhein, Germany), the glucagon receptor (GCGR) antagonist REMD2.59 was from REMD Bioptherapeutics (Camarillo, CA, USA) and pertussis toxin (PTX) was from Sigma (Gillingham, Dorset, UK). The Ca^2+^ channel blockers ω-agatoxin and isradipine, respectively blocking voltage-gated Ca^2+^ channels sensitive to ω-agatoxin (P/Q-type Ca^2+^ channels) and L-type Ca^2+^ channels, were from Alomone Labs (Jerusalem, Israel). The glucagon receptor antagonists (GRA) L-168049 and REMD2.59 were from Tocris Bioscience (Bristol, UK) and REMD Biotherapeutics Inc (Camarillo, CA, USA), respectively. Sitagliptin was obtained from Stratech Scientific (Ely, UK). The protein kinase A (PKA) inhibitor 8-Br-Rp-cAMPS was purchased from BioLog Life Science Institute (Bremen, Germany).

### Measurements of islet hormone secretion

Mouse islets were used acutely, except for studies with PTX, wherein islets were treated overnight with the toxin. Human islets were maintained in culture for up to 48 h in RPMI medium containing 10% (vol./vol.) FCS, 1% (vol./vol.) penicillin/streptomycin and 5 mmol/l glucose. Experiments were conducted as previously described [15]. Islets were incubated in 0.3 ml EC1 or EC2 media (ESM Table 2) supplemented with glucose and other reagents as indicated. Glucagon was determined by ELISA (Mercodia). The fractional glucagon release in isolated islets was 0.39±0.06 %/h (mean value ± SEM of 15 preparations using 150 mice). For comparison, glucagon secretion in the intact mouse pancreas perfused at the physiological rate (~0.3 ml/min) measured at 1 mmol/l glucose using the same assay was 86±17 pg/min (*n*=10) (i.e. 5.16±1.02 ng/h). Total pancreatic glucagon content was 1.16±0.06 μg (*n*=6). Thus, the rate of glucagon secretion normalised to the content in the perfused pancreas was estimated as 0.44±0.08%/h, in fair agreement with that obtained in the isolated islets.

Insulin and somatostatin were determined by RIA (Millipore and Diasource ImmunoAssays, respectively) as described previously [16, 17]. In these experiments, rates of release are expressed as % of contents unless otherwise indicated.

### Perfused mouse pancreas

Briefly, the aorta was ligated above the coeliac artery and below the superior mesenteric artery and then cannulated. The pancreas was perfused at 1.34 µl min^−1^ mg^−1^ pancreas weight using an Ismatec Reglo Digital MS2/12 peristaltic pump. Pancreatic weight was estimated from whole body weight as previously described [18, 19]. The perfusate was maintained at 37°C using a Warner Instruments temperature control unit TC-32 4B in conjunction with an in-line heater (Warner Instruments P/N 64-0102) and a Harvard Apparatus heated rodent operating table. The effluent was collected in intervals of 1 min into 96-well plates kept on ice and containing aprotinin. Samples were subsequently stored at −80°C pending analysis of glucagon content (using the Mercodia assay).

### Immunohistochemistry

Pancreatic islets were fixed overnight at +4°C using 4% (wt/vol.) paraformaldehyde and subsequently rinsed in PBS. Endogenous peroxidase activity was blocked with 0.3% hydrogen peroxide (vol./vol.) in methanol and non-specific immunoreactivity was inhibited by incubation with 5% normal swine serum. Islets were incubated overnight at 4℃ with rabbit anti-GCGR antibody (catalogue no. ab75240, 1:100 dilution; Abcam, Cambridge, UK) and mouse monoclonal anti-glucagon antibody (1:100 dilution, Sigma). Following washing, polyclonal goat anti-rabbit IgG coupled to horseradish peroxidase (1:100; Thermo Fisher; for GCGR) plus goat anti-mouse TRITC (1:100; Thermo Fisher; for glucagon) were applied for 30 min. Following more washing steps, tyramide signal amplification was used for the GCGR antibody using a kit according to the manufacturer’s instructions (Alexa Fluor 488 Tyramide Reagent; Thermo Fisher). Islets were then incubated with an Alexa 546-conjugated goat anti-mouse immunoglobulins (1:500 dilution; Thermo Fisher) at room temperature. Triton X-100 (0.01% vol./vol.; Sigma) and swine serum (1%) were present throughout the staining and washing processes. Islets were finally washed in PBS before scanned using a BioRad Radiance 2100 laser scanning confocal microscope controlled with LaserSharp software (version 4.3; BioRad, UK). Image resolution was 512×512 pixels through a 60× objective (Nikon). Negative controls (omission of primary antibody) were used to confirm primary and secondary antibody specificity. Glucagon- and GCGR-positive cells were manually counted in five mouse islets using Fiji (version 1.53t, National Institutes of Health, MD, USA, https://imagej.net/software/fiji/downloads). No immunoreactivity in pancreatic islets was observed with the ab75240 antibody in *Gcgr*^−/−^ mice [20].

### G protein dissociation studies (TRUPATH)

G protein dissociation was measured using the TRUPATH biosensor platform [21]. TRUPATH constructs were obtained from Addgene (kit no. 1000000163 [21]). The HEK293T cells (no. CRL-3216; from ATCC), for which routine mycoplasma testing was performed every 3 months, were grown in DMEM supplemented with 10% FBS and 1% antibiotic–antimycotic solution. Cells were transfected with equal amounts of GCGR, GαsS-, Gαi1-, Gαi2-, Gαi3-, GαoA-, GαoB- or Gαz-Rluc8, Gβ3, and Gγ1-, Gγ8- or Gγ9-GFP2 (as described in [21]), and pcDNA3.1(‑) using polyethyleneimine (ESM Table 3). After 24 h, cells were seeded at 50,000 cells/well of a 0.01% poly-l-lysine-coated white 96-well plate (Greiner) and grown overnight. Cells were washed with Hanks’ Balanced Salt Solution (Lonza) buffered with 20 mmol/l HEPES (Sigma) and supplemented with 0.1% BSA (Sigma) (wt/vol.). Cells were incubated in 5 μmol/l coelenterazine 400a (Nanolight Technologies) for 5 min. Ligands were added immediately prior to reading on a Mithras LB 940 multimode microplate reader (emission filters at 400 nm and 515 nm). The bioluminescence resonance energy transfer 2 (BRET2) ratio was calculated (λ515/λ400) and the time point corresponding to maximal dissociation was used to generate dose–response curves. pEC_50_ values were calculated using the ‘three parameter log(agonist) vs response’ model in GraphPad Prism9 (version 9.1.0; graphpad.com).

### PKA activity measurements

PKA activity was measured using AKAR3 as previously described [22]. The cells were continuously superfused at a rate of 60 μl/min with EC4 (ESM Table 2). Alpha cells were identified as those exhibiting a response to 10 μmol/l adrenaline (epinephrine) [23].

### Cytoplasmic free Ca^2+^ concentration imaging

For live-cell cytoplasmic free Ca^2+^ concentration ([Ca^2+^]_i_) imaging experiments, Gcg-GCaMP6f islets were immobilised to a poly-l-lysine-coated coverslip fixed in a custom-built imaging chamber filled with EC3 (ESM Table 2). [Ca^2+^]_i_ was measured in islets from mice expressing GCaMP6f in alpha cells. [Ca^2+^]_i_ imaging experiments were then performed using an inverted LSM 510 confocal microscope (Zeiss) controlled with ZEN Black (Zeiss), using a ×40/1.3 oil immersion objective. Time-lapse images were collected every 0.98 s with a frame size of 256×256 pixels, with the bath solution (EC3) perfused at a rate of 0.4 ml/min and heated to 37°C. GCaMP6f was excited by an argon laser (488 nm) and emission was collected at 510 nm.

[Ca^2+^]_i_ imaging videos were analysed using the Fiji imaging processing package. The mean fluorescence (*F*) of each region of interest was normalised to baseline signal (*F*_0_) and expressed as *F*/*F*_0_ before exporting into ClampFit (version 10.7; Molecular Devices, CA, USA), where baseline was corrected and AUC was calculated.

### Total internal reflection fluorescence microscopy imaging of granule mobility

Cells were imaged using total internal reflection fluorescence (TIRF) microscopy (AxioObserver Z1 with a ×100/1.45 objective [Carl Zeiss] and a diode-pumped solid state laser at 491 nm). For these measurements, EC1 was used (ESM Table 2). Images from cells infected with preproglucagon promoter (PPPG)-neuropeptide Y (NPY)-enhanced green fluorescent protein (EGFP) virus were recorded using an electron-multiplying charge-coupled device camera (Photometrics Evolve) using ZEN blue [24]. Single images of cells were acquired to measure the number of docked granules. Incoming and outgoing granules during visiting, docking and undocking were determined from multiple frame movies as described previously [25]. Candidate docking or undocking events were found manually as granules that approached the TIRF field with an axial component and became laterally confined for at least two frames. We defined docking as granules that remained confined for at least 1 min. Visitors were those granules that remained for <1 min after appearing at the plasma membrane. Undocking was defined as slow movement of a previously docked granule away from its docking site [25]. Granule density was calculated using a script that used the built-in ‘find maxima’ function in ImageJ (version 1.53c; http://rsbweb.nih.gov/ij) for spot detection [26].

### Plasma glucose and glucagon measurements

C57Bl6N mice were fasted for 5 h prior to the ITTs. GLP-1(9–36) (100 µg/kg body weight) was injected intraperitoneally before or with insulin as indicated. The relative high dose of GLP-1(9–36) was used because of high basal levels of the peptide. In the control experiments, mice were injected with the saline solvent (154 mmol/l NaCl). Tail-vein blood glucose and glucagon levels were monitored before and during injection of insulin (0.75 U/kg body weight in PBS; Actrapid; Novo Nordisk). Plasma glucagon was measured using a glucagon ELISA (10-1281-01; Mercodia, Uppsala, Sweden; CV<7%). Blood glucose levels were measured using a glucometer (Accu-Chek; Roche). Total GLP-1 was measured by ELISA (81508; Crystal Chem, Zaandam, the Netherlands; CV<10%).

In the experiments involving the use of REMD2.59, mice were injected intraperitoneally with the antagonist (5 mg/kg body weight in PBS) or vehicle 24 h prior to insulin-induced hypoglycaemia. Following the completion of the experiment, the mice were euthanised and the pancreases were resected and weighed. The pancreases were then homogenised, sonicated in acid ethanol and stored at 4°C for glucagon extraction.

### Statistical analysis

All data are reported, unless otherwise stated, as mean values ± SEM for the indicated number of experiments using islets from multiple mice. For hormone release studies, islets were isolated and pooled from 4–16 mice. These islets were then subdivided into groups of 12–20 size-matched islets. Each unique set of islets counted as an experiment with the experiments repeated on 2–4 days. Similarly, each unique group of human islets was treated as an experiment. Because the experiments were conducted over more than a decade by different investigators and using mice of different ages, glucagon secretion rates have been (for display) normalised to the mean rate of secretion at 1 mmol/l glucose (expressed as pg/islet × h). In the Ca^2+^ imaging and granule docking experiment, each cell represents an experiment making sure that cells from multiple donors/animals were used. For the in vivo experiments and the perfused pancreas, each mouse count represents an experiment. For two groupings, a *t* test was conducted. For multiple comparisons, one-way ANOVA was conducted. All statistical tests were performed using Graphpad Prism (version 9.1.0; graphpad.com). It was ascertained that the data were normally distributed.

## Results

### GLP-1(9–36) mimics glucagonostatic effect of GLP-1(7–36)

**Fig. 1:**
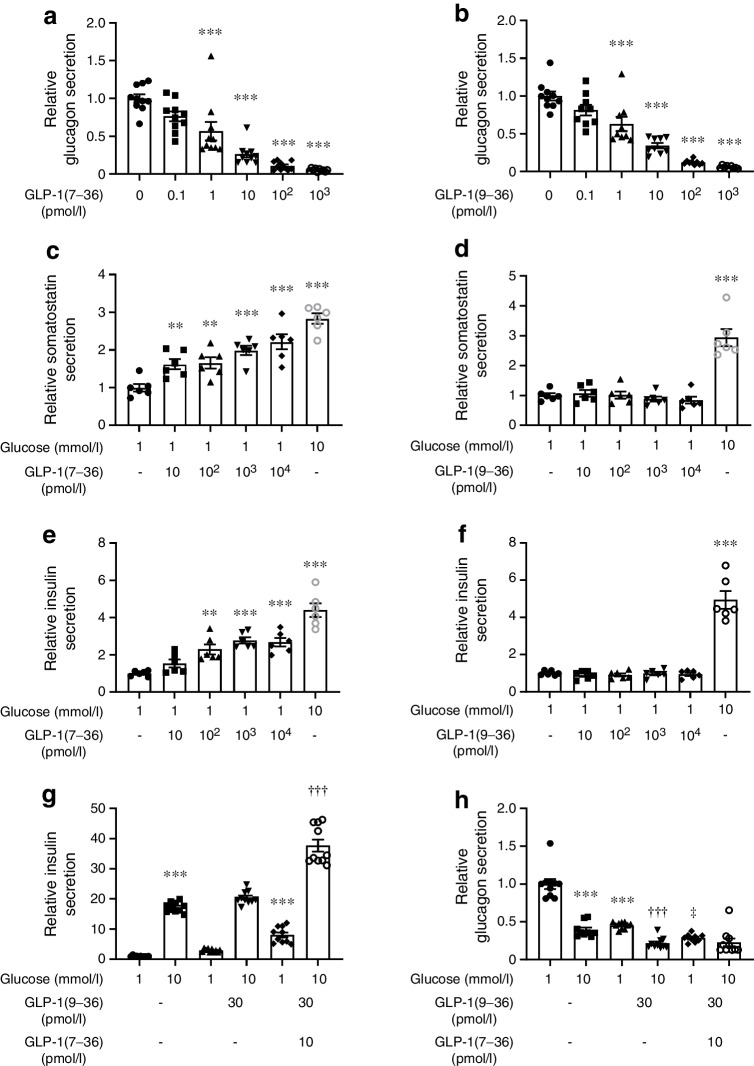
GLP-1 inhibits glucagon secretion. (**a**) Effect of increasing concentrations of GLP-1(7–36) on glucagon secretion in isolated mouse islets. Each data point represents a unique group of 12 islets isolated from 4–6 mice. Glucagon secretion has been normalised to that at 1 mmol/l glucose (1=17±0.36 pg islet^−1^ h^−1^). Rectangles and error bars represent mean values ± SEM. ***p<0.001 (one-way ANOVA followed by Tukey’s post hoc test). (**b**) As for (**a**) but using GLP-1(9–36) (1=10.0±0.4 pg islet^−1^ h^−1^). ***p<0.001. (**c**, **d**) As for (**a**, **b**) but effects on somatostatin secretion were measured. Each data point represents a unique group of 20 islets isolated from 4 mice. Somatostatin secretion has been normalised to that at 1 mmol/l glucose (1=0.074±0.007% and 0.055±0.004% of content/h in **c** and **d**, respectively). **p<0.01, ***p<0.001 (one-way ANOVA followed by Tukey’s post hoc test). (**e**, **f**) As for (**c**, **d**) but insulin secretion was measured (1=0.055±0.003% and 0.065±0.003% of content/h in **e** and **f**, respectively). **p<0.01, ***p<0.001 (one-way ANOVA followed by Tukey’s post hoc test). (**g**) Effects of GLP-1(9-36) and GLP-1(7-36) on insulin secretion at 1 and 10 mmol/l glucose. Insulin secretion has been normalised to that at 1 mmol/l glucose (1=29±2 pg islet^−1^ h^−1^). ***p<0.001 vs 1 mmol/l glucose; ^†††^*p*<0.001 vs 10 mmol/l glucose and 30 pmol/l GLP-1(9–36) (one-way ANOVA followed by Tukey’s post hoc test). Concentrations of GLP-1(9–36) and GLP-1(7–36) used at both the glucose concentrations. (**h**) As for (**g**) but glucagon secretion was measured (1=4.2±0.3 pg islet^−1^ h^−1^). ***p<0.001 vs 1 mmol/l glucose; ^†††^*p*<0.001 vs 10 mmol/l glucose alone; ^‡^*p*<0.05 vs 1 mmol/l glucose and 30 pmol/l GLP-1(9–36) (one-way ANOVA followed by Tukey’s post hoc test)

We measured glucagon secretion in isolated mouse pancreatic islets exposed to 1 mmol/l glucose, a condition mimicking systemic hypoglycaemia and thus stimulating glucagon secretion. GLP-1(7–36) produced a dose-dependent inhibition of hypoglycaemia-stimulated glucagon secretion (Fig. 1a). In this series of experiments, the half-maximal inhibitory concentration (IC_50_) was 2.5 pmol/l. Unexpectedly, the metabolite GLP-1(9–36) inhibited glucagon secretion almost as potently as GLP-1(7–36), with an IC_50_ of 4 pmol/l (Fig. 1b).

In separate experiments, GLP-1(7–36) produced a dose-dependent stimulation of somatostatin secretion that was detectable already at 10 pmol/l (Fig. 1c). Insulin secretion was also stimulated by GLP-1(7–36) but this effect only attained statistical significance at concentrations ≥100 pmol/l. No stimulatory effects of GLP-1(9–36) (10–10,000 pmol/l) on somatostatin or insulin secretion were observed (Fig. 1d). These data make it likely that the glucagonostatic effect of GLP-1(9–36) reflects a direct action rather than an indirect (paracrine) effect on the alpha cells. However, part of the glucagonostatic effect of GLP-1(7–36) might be exerted via somatostatin and/or insulin.

### Differential effects of GLP-1(7–36) and (9–36) on insulin and glucagon secretion

We compared the effects of GLP-1(7–36) and GLP-1(9–36) on insulin (Fig. 1e) and glucagon secretion (Fig. 1f) at 1 and 10 mmol/l glucose (to mimic the situation in mice during hypoglycaemia and normoglycaemia). Increasing glucose from 1 to 10 mmol/l stimulated insulin secretion >15-fold over basal. In mice fasted for 5 h, the total plasma GLP-1 concentration averaged 34±7 pmol/l (*n*=7), most of which can be expected to exist as GLP-1(9–36) [27]. In vitro, GLP-1(9–36) (30 pmol/l) alone did not increase insulin secretion when tested at either glucose concentration. However, the combination of GLP-1(7–36) (10 pmol/l) and (9–36) (30 pmol/l), to emulate what occurs postprandially [5], increased insulin secretion at 10 mmol/l glucose. We measured glucagon release in the same experiments. Increasing glucose from 1 to 10 mmol/l inhibited glucagon secretion by 60%. GLP-1(9–36) inhibited glucagon secretion by 55% at 1 mmol/l glucose and potentiated the glucagonostatic effect at 10 mmol/l glucose (−50% compared with 10 mmol/l glucose alone). The combination of GLP-1(7–36) and (9–36) was slightly more inhibitory than GLP-1(9–36) alone when tested at 1 mmol/l glucose but did not produce an additional inhibition at 10 mmol/l glucose.

We confirmed these observations in human islets. When tested at a concentration of 10 pmol/l, both GLP-1(7–36) and GLP-1(9–36) inhibited glucagon secretion at 1 mmol/l glucose by ~50% (ESM Fig. 1a, b). GLP-1(9–36) also exerted a glucagonostatic effect at 6 mmol/l glucose but not at 20 mmol/l glucose.

### GLP-1’s glucagonostatic effect is retained in Glp1r^−/−^ islets

**Fig. 2:**
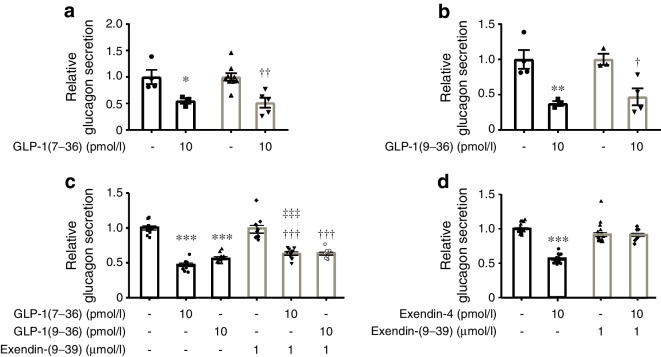
GLP-1’s glucagonostatic effect does not require *Glp1r*. (**a**) Effects of 10 pmol/l GLP-1(7–36) on glucagon secretion in wild-type (black) and *Glp1r*^−/−^ (grey) islets. Each data point represents a unique group of 12 islets isolated from 4 mice of each genotype. Glucagon secretion has been normalised to that at 1 mmol/l glucose (1=3.4±0.46 pg islet^−1^ h^−1^ and 2.6±0.2 pg islet^−1^ h^−1^ in wild-type and *Glp1r*^*−/−*^ islets, respectively). *p<0.05 vs control in wild-type islets; ^††^*p*<0.01 vs control in *Glp1r*^−/−^ islets. (**b**) As for (**a**) but testing 10 pmol/l GLP-1(9–36) and using 3 mice of each genotype (1=3.5±0.5 pg islet^−1^ h^−1^ and 2.2±0.17 pg islet^−1^ h^−1^ in wild-type and *Glp1r*^−/−^ islets, respectively). **p<0.05 vs control in wild-type islets; ^†^*p*<0.05 vs control in *Glp1r*^−/−^ islets. (**c**) Effects of 10 pmol/l GLP-1(7–36) and (9–36) on glucagon secretion in the absence and presence of the GLP-1R antagonist exendin(9–39) (1 μmol/l) as indicated. Each data point represents a unique group of 12 islets isolated from 8 mice. Glucagon secretion has been normalised to that at 1 mmol/l glucose (1=7.4±0.2 pg islet^−1^ h^−1^). ***p<0.001 vs 1 mmol/l glucose; ^†††^*p*<0.001 vs exendin(9–39); ^‡‡‡^*p*<0.001 vs 10 pmol/l GLP-1(9–36). (**d**) Effects of exendin-4 (10 pmol/l) in the absence and presence of exendin(9–39) (1 μmol/l) as indicated. Each data point represents a unique group of 12 islets isolated from 8 mice. Glucagon secretion has been normalised to that at 1 mmol/l glucose (1=3.88±0.12 pg islet^−1^ h^−1^). ***p<0.001. Statistical analyses in (**a**–**c**) were carried out by one-way ANOVA followed by Tukey’s post hoc test

We tested the effects of genetic ablation of *Glp1r* (encoding GLP-1 receptor [GLP-1R]) on insulin and glucagon secretion. Whereas GLP-1(7–36)’s insulinotropic effect was lost in *Glp1r*^−/−^ mouse islets (ESM Fig. 2a, b), the inhibitory effect of GLP-1(7–36) and (9–36) on glucagon secretion persisted (Fig. 2a,b). The mouse model used for these experiments is a general knockout and we can therefore discount the possibility that GLP-1 exerts its inhibitory action by paracrine effects secondary to activation of GLP-1Rs in delta and beta cells, suggesting that GLP-1’s glucagonostatic effect involves a receptor distinct from GLP-1R. These findings were confirmed using the GLP-1R antagonist exendin(9–39). In the presence of exendin(9–39), both GLP-1(7–36) and (9–36) retained their glucagonostatic effect (Fig. 2c) but it is notable that the inhibitory effect of GLP-1(7–36) (10 pmol/l) was reduced by exendin(9–39), indicating that part of GLP-1(7–36)’s action is mediated by GLP-1R (possibly via stimulation of somatostatin secretion; Fig. 1c). Exendin(9–39) itself did not affect glucagon secretion at 1 mmol/l glucose (Fig. 2c,d). The insulinotropic effects of GLP-1(7–36) are shared with the GLP-1R agonist exendin-4 [28], which is resistant to degradation by DPP-4 [29]. DPP-4, the enzyme catalysing the formation of GLP-1(9–36), is expressed and functionally active in isolated pancreatic islets [30]. Exendin-4 inhibited glucagon secretion. Unlike the effects of GLP-1(7–36) and (9–36), this effect was abolished in the presence of exendin(9–39) (Fig. 2d). We confirmed that exendin-4 potentiated insulin secretion at 6 mmol/l glucose by 100% in both mouse and human islets (not shown).

### GLP-1(9–36) inhibits glucagon secretion by activation of G_i/o_

**Fig. 3:**
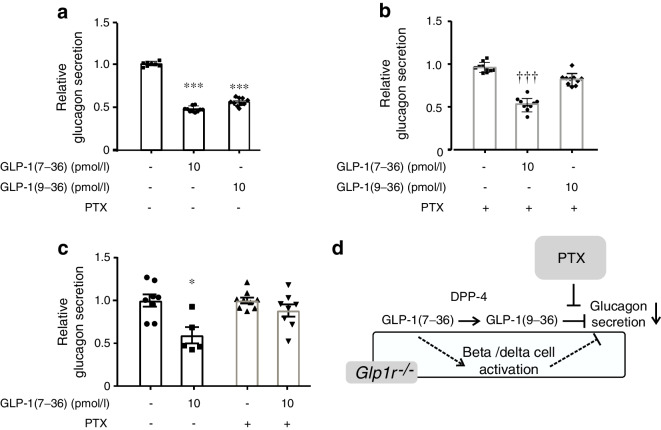
GLP-1(9–36) exerts its glucagonostatic effects by PTX-sensitive mechanisms. (**a**, **b**) Effects of GLP-1(7–36) and GLP-1(9–36) on glucagon release without (**a**) or with (**b**) overnight pretreatment with PTX (100 ng/ml) as indicated. Each data point represents a unique group of 12 islets isolated from 16 mice. Glucagon secretion has been normalised to that at 1 mmol/l glucose (1=10.7±0. pg islet^−1^ h^−1^). ***p<0.001 vs control; ^†††^*p*<0.001 vs 1 mmol/l glucose in PTX-treated islets. In (**a**) islets were cultured overnight without PTX. (**c**) Effects of 10 pmol/l GLP-1(7–36) on glucagon release in *Glp1r*^−/−^ islets under control conditions and after pretreatment with PTX. Each data point represents a unique group of 12 islets isolated from 10 mice. Glucagon secretion has been normalised to that at 1 mmol/l glucose (1=4.3±0.6 pg islet^−1^ h^−1^ and 5.7±0.95 pg islet^−1^ h^−1^ in the absence and presence of PTX, respectively. *p<0.05 vs no GLP-1(7–36) in control islets. (**d**) Schematic showing conversion of GLP-1(7–36) into GLP-1(9–36) and impact of PTX pretreatment. GLP-1(7–36) (and exendin-4), via activation of GLP-1Rs in beta and delta cells, inhibits glucagon secretion by a PTX-resistant paracrine mechanism (rectangle) that is lost following ablation of the GLP-1R (*Glp1r*^−/−^). Statistical analyses in (**a**–**c**) were carried out by one-way ANOVA followed by Tukey’s post hoc test

We hypothesised that the glucagonostatic effects of GLP-1(9–36) are mediated by activation of an inhibitory GTP-binding protein (G_i/o_). To test this, we pretreated islets with PTX [31]. In the absence of PTX, both GLP-1(7–36) and (9–36) inhibited glucagon secretion by ~50% (Fig. 3a). Following pretreatment of the islets with PTX, the inhibitory effect of GLP-1(9–36) was almost abolished but that of GLP-1(7–36) was unaffected (Fig. 3b). The inhibitory effect of GLP-1(7–36) on glucagon secretion that persists in islets from *Glp1r*^−/−^ mice was abolished by pertussis toxin (Fig. 3c). The glucagonostatic effect of GLP-1(7–36) that remains in the presence of exendin(9–39) could be abolished by the DPP-4 inhibitor sitagliptin [32] (ESM Fig. 3). Collectively, these data suggest that the *Glp1r*-independent effects of GLP-1(7–36) are exerted following its degradation to GLP-1(9–36) and is mediated by activation of G_i/o_. In addition, GLP-1(7–36) exerts *Glp1r*-dependent effects, which are prevented following genetic ablation of the receptor, possibly via paracrine signals originating from the beta and delta cells (Fig. 3d).

### GLP-1(9–36) inhibits PKA activity by a G_i/o_-dependent mechanism

**Fig. 4:**
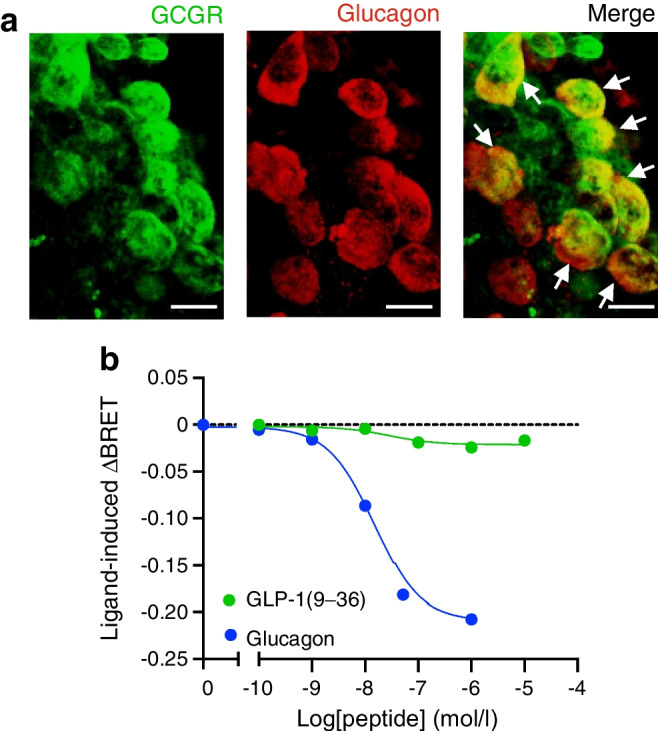
Glucagon receptors in alpha cells and their activation by GLP-1(9–36). (**a**) Glucagon receptor (GCGR) immunoreactivity in alpha cells. Double immunofluorescence staining of GCGR (green) and glucagon (red) in a mouse pancreatic islet. GCGR staining was merged with glucagon staining to test the co-localisation. GCGR and glucagon double-positive cells are indicated with white arrows. Scale bar, 10 μm. (**b**) Effects of increasing concentrations of glucagon or GLP-1(9–36) (logarithmic scale) on dissociation of the G_oA_ GTP-binding protein α-subunit from GCGRs expressed in HEK293T cells using the TRUPATH biosensor platform. Effects are expressed as the ligand-induced change in BRET (relative to that in the absence of any peptides; ΔBRET, *y*-axis) against concentration of glucagon or GLP-1(9–36) (*x*-axis). Data representative of 4 and 3 replicates for glucagon and GLP-1(9–36), respectively. See also ESM Table 4.

The GCGR can activate both stimulatory (G_s_) and inhibitory (G_i/o_) GTP-binding proteins [33–35] and may therefore mediate the PTX-sensitive effects of GLP-1(9–36). Published RNA-seq data of mouse and human alpha and beta cells indicate that expression levels of *Gcgr/GCGR* are much lower in alpha cells than in beta cells [36, 37]. Nevertheless, GCGR immunoreactivity was detected in 34±4% of the glucagon-positive alpha cells (as calculated from the images in Fig. 4a). Our data are in agreement with a recent report using the same antibody, the specificity of which is suggested by the loss of immunoreactivity in islets from *Gcgr*^*−/−*^ mice [20].

We determined the GTP-binding protein coupling of human GCGR expressed in HEK293T cells. GCGR potently coupled to both G_s_ and G_i/o_ proteins in response to glucagon (ESM Fig. 4a–f). Notably, GLP-1(9–36) increased dissociation of the inhibitory GTP-binding protein G_oA_ (Fig. 4b) with similar potency but lower efficacy (~10%) than glucagon (ESM Table 4).

**Fig. 5:**
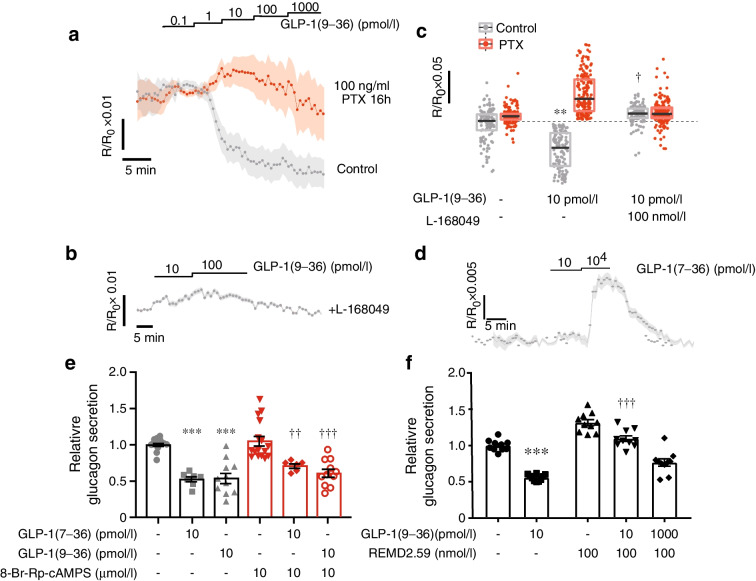
GLP-1(9–36) inhibits PKA activity and glucagon secretion by GCGR-dependent mechanism. (**a**) Effects of increasing concentrations of GLP-1(9–36) (staircase) on PKA activity in individual alpha cells in intact islets under control conditions (*n*=420 cells from 3 mice) and after pretreatment with PTX (*n*=785 cells from 3 mice). Data are mean values ± SEM. (**b**) PKA activity in alpha cells in response to 10 and 100 pmol/l GLP-1(9–36) in the presence of 100 nmol/l of L-168049 (*n*=420 cells from 3 mice). In (**a**, **b**) responses have been normalised to basal conditions prior to the addition of the agonists. (**c**) Box plots of changes in PKA activity in response to 10 pmol/l GLP-1(9–36) under control conditions and after pretreatment with PTX (*n*=785 cells from 3 mice) or in the presence of 100 nmol/l of L-168049. **p<0.01 vs basal level (evaluated by Friedman ANOVA, Nemenyi post hoc test); ^†^*p*<0.05 vs GLP-1(9–36) in the absence of L-168049 (Kruskal–Wallis ANOVA, Nemenyi’s post hoc test). Black lines represent medians and the boxes indicate first and third quartiles. (**d**) As for (**b**) but testing the effects of 10 pmol/l and 10,000 pmol/l GLP-1(7–36) (*n*=420 cells from 3 mice). (**e**) Effects of GLP-1(7–36) and (9–36) on glucagon secretion in the absence (black) or presence (red) of 8-Br-Rp-cAMPS (10 μmol/l) as indicated. Each data point represents a unique group of 12 islets isolated from 10–11 mice. Glucagon secretion has been normalised to that at 1 mmol/l glucose (1=1.8±0.3 pg islet^−1^ h^−1^). ***p<0.001 vs control in the absence of 8-Br-Rp-cAMPS; ^††^*p<*0.01 and ^†††^*p*<0.001 vs control in the presence of 8-Br-Rp-cAMPS (one-way ANOVA and Tukey’s post hoc test). (**f**) Effects of 10 pmol/l and 1000 pmol/l GLP-1(9–36) on glucagon secretion in the absence (black) or presence (red) of 100 nmol/l of the monoclonal antibody/antagonist REMD2.59. Each data point represents a unique group of 12 islets isolated from 6 mice. Glucagon secretion has been normalised to that at 1 mmol/l glucose (1=7.4±0.1 pg islet^−1^ h^−1^). ***p<0.001 vs no GLP-1(9–36); ^†††^*p*<0.001 vs 10 pmol/l GLP-1 in the absence of REMD2.59 (one-way ANOVA followed by Tukey’s post hoc test)

There are multiple targets of G_i/o_ activation in alpha cells [38]. GLP-1(9–36) produced a dose-dependent decrease in cAMP/PKA activity that was maximal at concentrations >10 pmol/l (Fig. 5a,c). Pretreatment with PTX abolished the inhibitory effect of GLP-1(9–36) on PKA activity. Likewise, GLP-1(9–36) was without effect in the presence of the GCGR antagonist (GRA) L-168049 (Fig. 5b,c). Collectively, these data suggest that exogenous GLP-1(9–36) inhibits cAMP/PKA by a GCGR- and G_i/o_-dependent mechanism.

Unlike GLP-1(9–36), GLP-1(7–36) (10 pmol/l) had no inhibitory effect on PKA activity in alpha cells and when tested at 10,000 pmol/l (a concentration used in many earlier studies; e.g. [39, 40]) actually increased cAMP/PKA activity (Fig. 5d). Consistent with these data, the glucagonostatic effect of physiological levels of GLP-1(7–36) was not affected by the PKA inhibitor 8-Br-Rp-cAMPS (Fig. 5e), at variance with what was reported previously for high concentrations of the peptide in both mouse and human islets [41, 42].

### GRAs reverse GLP-1(9–36)’s glucagonostatic effect

We used REMD2.59, a human monoclonal antibody and competitive GRA [43, 44] that does not bind to GLP-1Rs [45], to inactivate the GCGRs. In the presence of REMD2.59, glucagon secretion in the presence of 10 pmol/l and 1000 pmol/l GLP-1(9–36) averaged 110±4% and 76±5% of that at 1 mmol/l glucose, respectively (Fig. 5f). On its own, REMD2.59 had a small stimulatory effect on glucagon secretion at 1 mmol/l glucose alone (~30%). In mouse islets, the glucagonostatic effect of GLP-1(9–36) was also abolished by the GRA L-168049 [46] (ESM Fig. 5a). Similar antagonistic effects of GRAs on GLP-1(9–36)’s glucagonostatic effects were observed in human islets using the antagonists desHis^1^Pro^4^Glu^9^ glucagon (peptide N) or desHis^1^Pro^4^Glu^9^Lys^12^PAL-glucagon (peptide R) [47] (ESM Fig. 5b).

### GLP-1(9–36) inhibits glucagon secretion equally regardless of stimulus

**Fig. 6:**
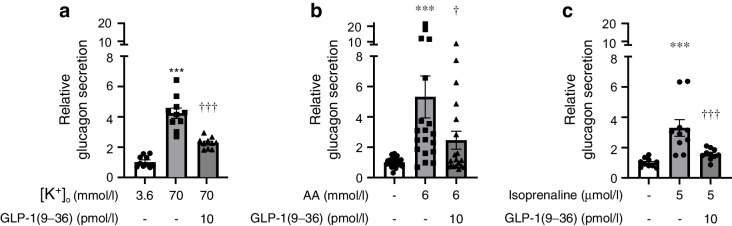
GLP-1(9–36) inhibits both depolarisation- and agonist-induced glucagon secretion. (**a**) Glucagon secretion at 3.6 or 70 mmol/l extracellular K^+^ ([K^+^]_o_) in the absence or presence of GLP-1(9–36) as indicated. Each data point represents a unique group of 12 islets isolated from 6 mice). Glucagon secretion has been normalised to that at 1 mmol/l glucose (1=16.9±1.9 pg islet^−1^ h^−1^). ***p<0.001 vs 1 mmol/l glucose at 3.6 mmol/l [K^+^]_o_; ^†††^*p*<0.001 vs 70 mmol/l [K^+^]_o_ alone. (**b**) Glucagon secretion at 1 mmol/l glucose in the absence or presence of a cocktail of AAs (2 mmol/l each of glutamine, alanine and arginine) and GLP-1(9–36) as indicated. Each data point represents a unique group of 12 islets isolated from 10 mice. Glucagon secretion has been normalised to that at 1 mmol/l glucose (1=36.5±7.3 pg islet^−1^ h^−1^). ***p<0.001vs no AAs; ^†^*p*<0.05 vs 6 mmol/l AA alone. (**c**) As for (**b**) but testing the effects of isoprenaline (1=11±1.9 pg islet^−1^ h^−1^; 9 mice). ***p<0.001 vs 1 mmol/l glucose; ^†††^*p*<0.001 vs isoprenaline. Statistical significance in (**a**–**c**) was estimated using one-way ANOVA with Tukey’s post hoc test

The finding that GLP-1(9–36) retains its glucagonostatic capacity in the presence of 8-Br-Rp-cAMPS suggests that this action is not mediated by inhibition of PKA. Other targets/mechanisms must therefore be considered. Glucagon secretion occurs by exocytosis of secretory granules triggered by Ca^2+^ entry via voltage-gated calcium channels (VGCCs) [48]. We explored whether GLP-1(9–36) inhibits glucagon secretion by influencing Ca^2+^ entry/exocytosis. Opening of the VGCCs evoked by membrane depolarisation, produced by increasing extracellular K^+^ ([K^+^]_o_) to 70 mmol/l, stimulated glucagon secretion by over threefold, and this response was reduced by 60% by GLP-1(9–36) (Fig. 6a). GLP-1(9–36) produced a similar (−66%) inhibition of amino acid (AA; 6 mmol/l)-induced glucagon secretion; the AAs had produced a fourfold increased glucagon secretion through increasing action potential firing with resultant opening of VGCCs [49] (Fig. 6b). Adrenaline (via activation of β-adrenoceptors, elevation of cAMP and mobilisation of intracellular [Ca^2+^]_i_) is a powerful glucagon secretagogue [22]. The β-adrenoceptor agonist isoprenaline stimulated glucagon secretion >2.2-fold, and this response was reduced 75% by GLP-1(9–36) (Fig. 6c).

### GLP-1(9–36) depletes docked granules pool

**Fig. 7:**
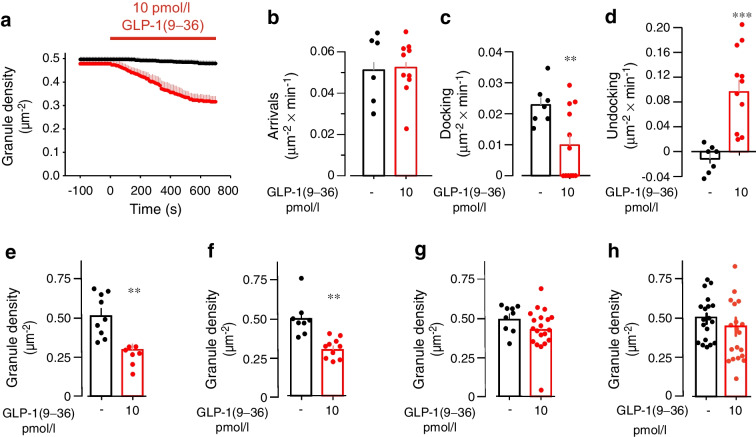
GLP-1 leads to undocking of secretory granules in alpha cells. (**a**) Docked granule density measured in mouse alpha cells in the absence (black) or presence (red) of GLP-1(9–36) (10 pmol/l). When tested, GLP-1(9–36) was included in the superfusion medium as indicated by the horizontal line. Data are mean values ± SEM of 7 cells from 3 mice for control and 12 cells from 4 mice for GLP-1(9–36). (**b**–**d**) Number of granules arriving (**b**), docking (**c**) and undocking (**d**) in the absence or presence of GLP-1(9–36) during 15 min in the experiments summarised in (**a**). Data in bar graphs represent mean values ± SEM superimposed on individual data points. (**e**–**h**) Granule density measured in the absence or presence of GLP-1(9–36) under control conditions (**e**), in the presence of exendin(9–39) (100 nmol/l) (**f**), with the GCGR antagonist L-168049 (100 nmol/l) (**g**) and after pretreatment with PTX (100 ng/ml) for 16 h (**h**). Granule density was measured 15 min after addition of GLP-1(9–36). Data points correspond to individual cells obtained from at least 3 different mice. Cells were pretreated with exendin(9–39) and L-168049 for 15 min before measurements commenced. Data were normalised to membrane area. **p<0.01, ***p<0.001 vs no GLP-1(9–36) in each panel (Student’s *t* test)

The fact that GLP-1(9–36) remains glucagonostatic regardless of whether glucagon secretion is triggered by low glucose alone, membrane depolarisation or receptor-induced elevation of cAMP suggests it may act late in the secretory process, possibly at the level of exocytosis itself. We used total TIRF microscopy to study the mean near-membrane granule trafficking [24]. Under control conditions (1 mmol/l glucose alone), the near-membrane granule density in alpha cells was ~0.5 μm^−2^ (Fig. 7a). GLP-1(9–36) produced a time-dependent 40% reduction of the number of docked granules, an effect that was maximal after 10 min. Given that GLP-1(9–36) inhibits glucagon secretion, it is unlikely that the reduction in the number of docked granules reflects stimulation of exocytosis. Indeed, under control conditions (1 mmol/l glucose alone), the decrease in granule density was <4%. GLP-1(9–36) instead reduced granule density by inhibiting granule docking and stimulating granule undocking, while not affecting the rate of arrival (Fig. 7b–d). The capacity of GLP-1(9–36) to reduce the number of docked granules was resistant to the GLP-1R antagonist exendin(9–39) (Fig. 7e,f) but was prevented by the GRA L-168049 (Fig. 7g) or overnight pretreatment with PTX (Fig. 7h).

### GLP-1(9–36) inhibits P/Q-type Ca^2+^ channels

**Fig. 8:**
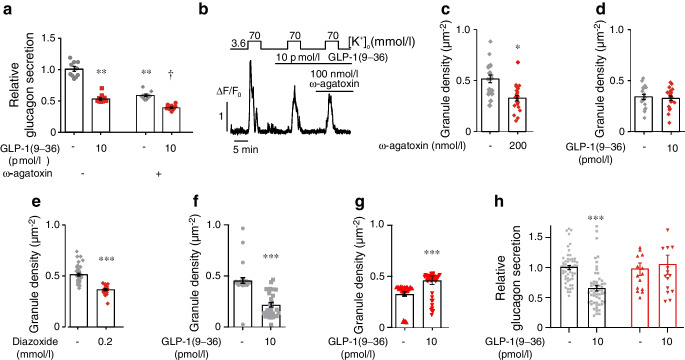
Impact of P/Q-type Ca^2+^ channel inhibition and type 2 diabetes on GLP-1(9–36)-induced granule undocking. (**a**) Glucagon secretion measured at 1 mmol/l glucose in the absence and presence of 10 pmol/l GLP-1(9–36) under control conditions (without ω-agatoxin) and in the presence of ω-agatoxin (200 nmol/l) as indicated. Note that the blocker, when added as indicated, was present in both the absence and presence of GLP-1(9–36). Each data point represents a unique group of 12 islets isolated from 6 mice. Glucagon secretion has been normalised to that at 1 mmol/l glucose (1=6.0±0.2 pg islet^−1^ h^−1^). **p<0.01 vs 1 mmol/l glucose; ^†^*p*<0.05 vs 1 mmol/l glucose and ω-agatoxin (one-way ANOVA followed by Dunnet’s post hoc test). (**b**) Depolarisation-evoked increases in [Ca^2+^]_i_ elicited by increasing extracellular K^+^ ([K^+^]_o_) from 3.6 to 70 mmol/l in the presence of 10 μmol/l isradipine (to block L-type Ca^2+^ channels). Under these experimental conditions depolarisation-induced Ca^2+^ entry will reflect P/Q-type Ca^2+^ channel activity. GLP-1(9–36) (10 pmol/l) and ω-agatoxin were included in the medium as indicated (*n*=139 cells from 4 mice). (**c**) Granule density measured in the absence or presence of ω-agatoxin (200 nmol/l). *p<0.05. (**d**) Effects of GLP-1(9–36) in the presence of ω-agatoxin (200 nmol/l). (**e**) As for (**c**) but testing the effect of diazoxide (0.2 mmol/l). Control cells were incubated in the presence of 0.1% DMSO (solvent used for diazoxide). ***p<0.001 vs 1 mmol/l glucose (Student’s *t* test). (**f**) Granule density measured in human alpha cells in the absence or presence of 10 pmol/l GLP-1(9–36) (measured after 15 min). Mean values ± SEM of 28 GLP-1(9–36)-treated and 27 control alpha cells from 3 donors. ***p<0.001 vs control (Student’s *t* test). (**g**) As for (**f**) but in alpha cells from donors with type 2 diabetes. Mean values ± SEM of 43 GLP-1(9–36)-treated and 31 control cells from two donors. ***p<0.001 vs control. (**h**) Effects of GLP-1(9–36) on glucagon secretion in islets from cadaveric healthy donors. Each data point represents a unique group of 12 islets isolated from 13 healthy donors (black bars and grey symbols) and 3 donors with type 2 diabetes (red bars and symbols). Glucagon secretion has been normalised to that at 1 mmol/l glucose (1=7.01±0.91 and 2.41±0.62 pg islet^−1^ h^−1^ from experiments carried out on islets from donors without and with type 2 diabetes, respectively). ***p<0.001 vs no GLP-1(9–36) in islets from healthy donors (one-way ANOVA with Dunnett’s post hoc test)

Glucagon secretion evoked by low glucose concentrations is mediated by activation of the ω-agatoxin-sensitive P/Q-type Ca^2+^ channels [50]. Indeed, ω-agatoxin inhibited glucagon secretion nearly as strongly as GLP-1(9–36) (Fig. 8a). GLP-1(9–36) reduced depolarisation-induced P/Q-type Ca^2+^ channel-mediated Ca^2+^ entry by 34±4% (*p*=0.001) (Fig. 8b). Addition of ω-agatoxin in the presence of GLP-1(9–36) did not produce any further inhibition (−8±4%; *p*=0.26). ω-Agatoxin reduced the number of docked granules in alpha cells to the same extent as GLP-1(9–36) (Fig. 8c) and GLP-1(9–36) had no additive effect on granule docking in the presence of the blocker (Fig. 8d). The K_ATP_ channel activator diazoxide (200 μmol/l) also reduced the submembrane granule density by ~40% (Fig. 8e).

### Effects of GLP-1(9–36) on granule docking and glucagon in human alpha cells and impact of type 2 diabetes

In human alpha cells from healthy donors, GLP-1(9–36) (10 pmol/l) reduced the number of docked granules by 52% (Fig. 8f) whereas in alpha cells from donors with type 2 diabetes it increased docking by 40% (9–36) (Fig. 8g). This correlated with the loss of GLP-1(9–36)-induced suppression of glucagon release in islets from donors with type 2 diabetes (Fig. 8h).

### Effects of GLP-1(9–36) in vivo

**Fig. 9:**
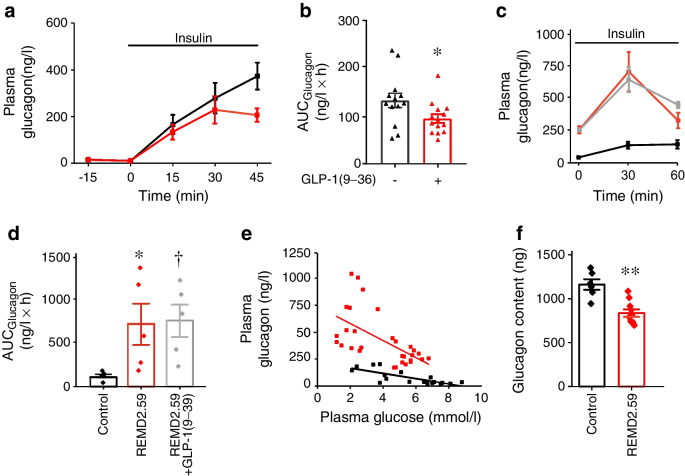
Effects of GLP-1(9–36) on plasma glucose and glucagon secretion during insulin-induced hypoglycaemia. (**a**) Plasma glucagon measured in mice with (red squares) or without (black squares) injection of GLP-1(9–36) (100 µg/kg body weight; at *t*=−15 min). GLP-1(9–36) was injected intraperitoneally and samples were taken at indicated times. At *t*=0 min, insulin (0.75 U/kg i.p.) was injected. Data are mean values ±SEM of 13 or 14 mice. (**b**) Dot plots of glucagon AUCs measured during 45 min following injection of insulin at *t*=0 min and later in (**a**) in the absence (black triangles) or presence (red triangles) of exogenous GLP-1(9–36). *p<0.05 vs control by Student’s *t* test. (**c**) Changes in plasma glucagon during insulin-induced hypoglycaemia (0.75 U/kg body weight i.p.) under control conditions (black squares/lines) and in mice pretreated with REMD2.59 with (grey squares/lines) or without (red squares/lines) pre-injection of GLP-1(9–36) (100 µg/kg body weight). (**d**) Dot plots of the AUCs of data in (**c**) under the indicated conditions. *p=0.05 and ^†^*p*<0.05 vs no REMD2.59 with/without GLP-1(9–36) control. (**e**) Relationship between plasma glucose and glucagon in vivo measured in (**d**). Data in REMD2.59-treated mice with/without GLP-1(9–36) were pooled. The black and red lines represent linear fits to the data points under control conditions (*r*=−0.77) and after pretreatment with REMD2.59 (*r*=−0.59; *p*<0.011 vs no REMD2.59). (**f**) Whole-pancreas glucagon content in control mice and mice pretreated with REMD2.59. **p<0.01 (Student’s *t* test)

We tested the effects of exogenous GLP-1(9–36) in vivo in mice (ESM Fig. 6a, b). GLP-1 levels increased promptly from a basal 30 pmol/l to a peak of 400 pmol/l and then declined exponentially but remained at >100 pmol/l for >45 min (probably reflecting the degradation by neutral endopeptidases [51]). The studies on isolated islets suggest that GLP-1(9–36)’s glucagonostatic capacity is particularly strong at low glucose. Its effects in vivo were therefore tested during insulin-induced hypoglycaemia (glucose lowered to ~3 mmol/l). Under normoglycaemic conditions (prior to insulin injection), basal plasma glucagon was <10 ng/l and was not affected by GLP-1(9–36). Hypoglycaemia triggered a large (>30-fold) increase in plasma glucagon, an effect that was slightly reduced by GLP-1(9–36) (Fig. 9a). When expressed as the AUC, GLP-1(9–36) produced a small decrease (~30%) during hypoglycaemia (Fig. 9b). No glucagonostatic effect (*p*=0.53) was detected when insulin and GLP-1(9–36) were administered simultaneously (ESM Fig. 6c).

We reasoned that high endogenous circulating levels of GLP-1(9–36) limit the glucagonostatic action of exogenous GLP-1(9–36). We tested insulin-induced hypoglycaemia in mice pretreated with a GRA. REMD2.59 reduced basal plasma glucose by ~2 mmol/l (ESM Fig. 6d) and increased circulating glucagon by 700% (Fig. 9c,d). Notably, GLP-1(9–36) had no glucagonostatic effect in the presence of REMD2.59. We determined the relationship between plasma glucose and glucagon in the absence and presence of REMD2.59 (Fig. 9e) and obtained slopes of −24±3 ng/mmol and −80±21 ng/mmol in the absence and presence of REMD2.59, respectively (*p*=0.0115). This corresponds to an increase of ~225% of plasma glucagon when corrected for the change in glucose concentration. Both relationships intercepted the *x*-axis at ~9 mmol/l glucose, close to the normal plasma glucose concentrations in fasted mice (ESM Fig. 6b, d). Thus, REMD2.59 principally stimulates glucagon secretion under hypoglycaemic conditions. The REMD2.59-induced increase in plasma glucagon correlated with a 27% reduction in pancreatic glucagon content (Fig. 9f). There was a negative correlation between pancreatic glucagon content and basal plasma glucagon (ESM Fig. 6e).

GRAs have been reported to increase circulating glucagon in vivo by hepatic hyperaminoacidaemia [52, 53]. We tested the effects of a cocktail of 3 and 6 mmol/l AAs. When applied at 1 mmol/l glucose, 3 mmol/l AAs resulted in a transient (5 min) stimulation of glucagon secretion. After the initial stimulation, glucagon secretion returned to the pre-stimulatory level and subsequently raising the AAs to 6 mmol/l was without stimulatory effect (ESM Fig. 6f).

## Discussion

**Fig. 10:**
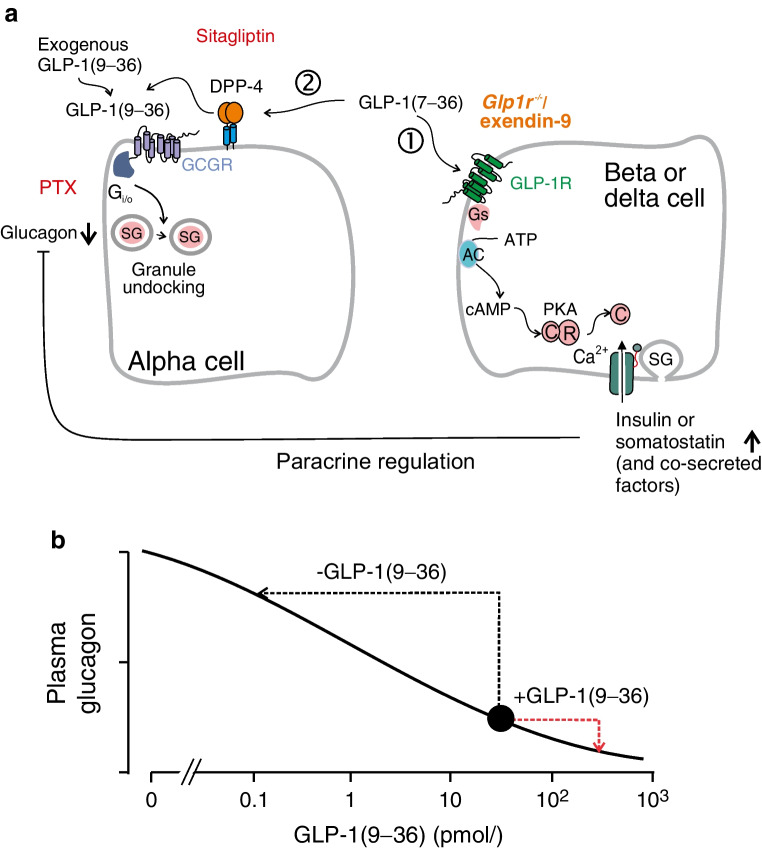
Regulation of glucagon secretion by physiological concentrations of GLP-1(7–36) and (9–36) via paracrine and intrinsic alpha cell mechanisms. (**a**) GLP-1(7–36) inhibits glucagon secretion by dual effects (indicated by 1 and 2). (1) Activation of GLP-1Rs in beta and delta cells stimulates the release of paracrine inhibitors of glucagon secretion. This mechanism is suppressed by pharmacological [using exendin(9–39)] or genetic inactivation of the GLP-1R. (2) GLP-1(9–36), generated by (DPP-4-mediated) degradation of GLP-1(7–36), results in activation of an inhibitory GTP-binding protein (G_i/o_), culminating (via undocking of secretory granules [SG] below the plasma membrane) in suppression of glucagon secretion. This mechanism is not affected by genetic/pharmacological inactivation of the GLP-1R but is sensitive to pertussis toxin. It is not activated by exendin-4, which is more resistant to DPP-4-induced degradation than GLP-1(7–36). GLP-1(7–36) will activate both (1) and (2) but exogenous GLP-1(9–36) will only activate (2). (**b**) Concentration-dependent inhibition of glucagon secretion by GLP-1(9–36). Because of high circulating GLP-1(9–36) levels, administration of high exogenous GLP-1(9–36) will only have a marginal additional glucagonostatic effect (red arrow). GRAs lead to a large increase in circulating glucagon by reversing the glucagonostatic effects of endogenous GLP-1(9–36) (black arrow)

We show that the GLP-1 metabolite GLP-1(9–36), previously assumed to lack biological activity, exerts a strong glucagonostatic effect both in vivo and in vitro. GLP-1(9–36)’s glucagonostatic effect operates in parallel with that of GLP-1(7–36), as illustrated schematically in Fig. 10a. According to this model, GLP-1(7–36) (at least at physiological concentrations; c.f. [42]) principally regulates glucagon secretion by paracrine mechanisms [54] resulting from activation of the GLP-1Rs in beta and delta cells. GLP-1(9–36), formed by the removal of the two N-terminal residues by DPP-4, leads to the activation of an inhibitory G protein (G_i/o_) and suppression of glucagon secretion. GLP-1(9–36) has a much longer t½ in circulation than GLP-1(7–36) [55] and the two peptides may regulate systemic metabolism with different kinetics following their release from the gut.

Genetic ablation of GLP-1Rs in alpha cells results in glucose intolerance in vivo and suppression of glucagon secretion in isolated islets at low glucose concentrations [56]. These observations raise the possibility the ligand GLP-1(7–36) itself, by increasing intracellular cAMP, exerts a stimulatory rather than an inhibitory effect on glucagon secretion [57]. Following its degradation, the glucagonotropic effect of GLP-1(7–36) will be superseded by the inhibitory effect of the metabolite GLP-1(9–36). The identity of the membrane receptor that mediates the latter effect remains to be conclusively established but our data suggest it is distinct from the GLP-1R. There have been multiple reports that GLP-1(9–36) exerts effects on the central nervous system, cardiovascular system, gastrointestinal tract, liver and muscle that persist in the absence of GLP-1R [55] and the existence of an alternative GLP-1R has therefore been inferred. However, attempts to identify it have failed and there is no obvious candidate in the genome [58, 59]. Our findings suggest that the GLP-1R-independent effects of GLP-1 may involve the activation of GCGRs. Although *Gcgr* is expressed at very low levels, the immunocytochemistry suggests that the protein is present in alpha cells (although below the detection level in 66% of the alpha cells). It is possible that the transcription of the gene is periodic [60] and that the short-lived mRNA (minutes/hours [61]) may thus be absent most of the time, whereas the long-lived protein (days [62]) remains present and functional. Indeed, the immunocytochemical data are supported by the functional data using a highly specific antagonist (REMD2.59) [43, 44]. The response to GLP-1(9–36) reported by the TRUPATH assay may seem minute. However, this assay measures the coupling between G protein-coupled receptors (GPCRs) and individual G proteins in an experimental system; under physiological conditions in alpha cells, downstream signalling cascades might amplify small signals. Accordingly, not many receptors need to be occupied to elicit the maximum inhibitory response. This concept also explains how activation of G_i/o_ by GLP-1(9–36) supersedes the stimulatory effect of G_s_ activation by β-adrenoceptor and GLP-1R activation. It is notable that neither the GLP-1R antagonist exendin(9–39) nor the GRA REMD2.59 had any (major) effect on glucagon secretion at 1 mmol/l glucose, making it unlikely that glucagon secretion is under significant autocrine control by glucagon or GLP-1(7–36) [63, 64] from alpha cells. It remains to be elucidated exactly how the GCGRs can respond to GLP-1(9–36) in the presence of the high intra-islet glucagon levels.

Previous studies have failed to document any glucagonostatic action of GLP-1(9–36) in vivo [7–9], seemingly at variance with the findings reported here. However, three factors may explain this discrepancy. First, the effect is glucose-dependent and GLP-1(9–36) exerts its predominant effect under hypoglycaemic conditions. Second, GLP-1(9–36) acts by granule undocking, a process that develops over 5–10 min and its glucagonostatic effect is therefore delayed. Third, because of high circulating GLP-1 levels, glucagon secretion will be under tonic suppression in vivo. From the dose–inhibition curves established in vitro, circulating GLP-1(9–36) (~30 pmol/l) can be expected to inhibit glucagon secretion by up to 80%, making it difficult to observe any additional suppression by exogenous administration (especially under normoglycaemic conditions when glucagon secretion is already strongly reduced) (schematic Fig. 10b). Conversely, reversal of GLP-1(9–36)’s glucagonostatic effect might explain the dramatic elevation of circulating glucagon observed after pharmacological/genetic inhibition of the GCGRs [43, 53, 65, 66], an effect that has been attributed to AA-induced stimulation of alpha cell proliferation. However, the acute effects of GRAs on circulating AAs are small (+25% [66]); AAs only transiently stimulate glucagon secretion and yet GRA treatment results in a dramatic elevation of circulating glucagon without increasing pancreatic glucagon content. Collectively, these observations militate against the idea that AA-induced alpha cell proliferation accounts for the high circulating levels of glucagon observed acutely upon administration of GRAs, an effect which we instead attribute to the removal of GLP-1(9–36)’s suppressor effect. Comparing the slopes of the relationships between plasma glucose and glucagon in the absence and presence of REMD2.59 suggests that glucagon secretion in vivo is reduced by 70%. From the dose–inhibition curves obtained in vitro we can estimate that this equates to a GLP-1(9–36) concentration of 30 pmol/l, in remarkably good agreement with the plasma GLP-1 concentration observed in vivo. We therefore propose that GLP-1(9–36) plays an important and previously unrecognised role as a systemic inhibitor of glucagon secretion (but not the only one). This concept by no means is incompatible with the finding that inhibition of GCGR signalling also results in alpha cell proliferation but we emphasise that the latter effect operates on a much longer time scale than the acute effect we now describe (days/weeks rather than minutes/hours). If anything, the marked elevation of circulating glucagon we observed following overnight pretreatment with REMD2.59 was associated with a reduction of pancreatic glucagon content.

The finding that GLP-1(9–36) acts by reducing the number of docked granules provides a simple and unifying explanation for its capacity to inhibit glucagon secretion regardless of whether it is evoked by low glucose, membrane depolarisation or a β-adrenergic agonist. The observations that both ω-agatoxin and diazoxide mimicked the effect of GLP-1(9–36) on granule docking suggest that Ca^2+^ influx via P/Q-type Ca^2+^ channels during alpha cell electrical activity [60] promotes granule docking in alpha cells.

In type 2 diabetes, the capacity of GLP-1(9–36) to deplete the docked pool of granules in alpha cells and suppress glucagon secretion was abolished. Type 2 diabetes is associated with elevated circulating levels of glucagon, especially when related to plasma glucose levels [67]. Our data suggest that reduced capacity of GLP-1(9–36) to exert its glucagonostatic effect might contribute to this defect and exacerbate the hyperglycaemia caused by impaired insulin secretion.

### Supplementary Information

Below is the link to the electronic supplementary material.

Supplementary file1 (PDF 624 KB)

## Data Availability

All data from this study are presented in the published article and the supplementary material. Additional information is available from the corresponding author upon request.

## Abbreviations

AA: Amino acid
[Ca^2+^]_i_: Cytoplasmic free Ca^2+^ concentration
DPP-4: Dipeptidyl peptidase 4
EGFP: Enhanced green fluorescent protein
GCGR: Glucagon receptor
GLP-1: Glucagon-like peptide 1
GLP-1R: GLP-1 receptor
GPCR: G protein-coupled receptor
GRA: Glucagon receptor antagonist
IC_50_: Half-maximal inhibitory concentration
NPY: Neuropeptide Y
PKA: Protein kinase A
PPPG: Preproglucagon promoter
P/Q-type Ca^2+^ channel: Voltage-gated Ca^2+^ channel sensitive to ω-agatoxin
PTX: Pertussis toxin
TIRF (microscopy): Total internal reflection fluorescence (microscopy)
VGCC: Voltage-gated calcium channel
