# Supplementary material for: GLP-1 metabolite GLP-1(9–36) is a systemic inhibitor of mouse and human pancreatic islet glucagon secretion
Source: Diabetologia. 2023 Dec 21;67(3):528–46. doi: 10.1007/s00125-023-06060-w (PMC10844371; doi:10.1007/s00125-023-06060-w)
Supplement: Supplementary file 1 — Supplementary file1 (PDF 624 KB) [file 125_2023_6060_MOESM1_ESM.pdf]

ELECTRONIC SUPPLEMENTARY MATERIAL

GLP-1 metabolite GLP-1(9-36) is a systemic inhibitor of mouse and human pancreatic islet glucagon secretion

Legends to ESM Figures

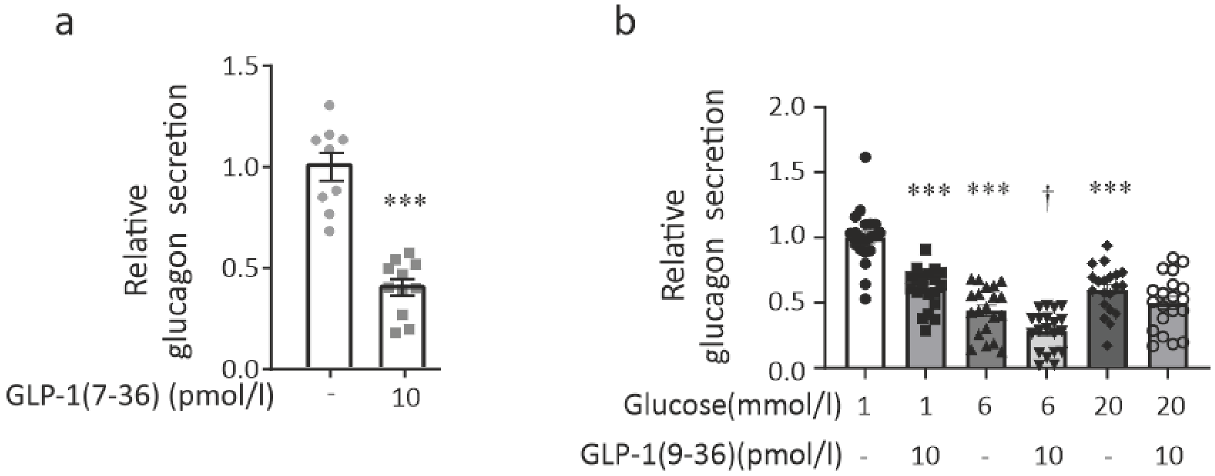

ESM Fig. 1 Regulation of glucagon secretion by GLP-1(7-36) and (9-36) in human islets. **(a)** Effects of 10 pmol/l GLP-1(7-36) on glucagon secretion in human islets. Each data point represents a unique group of 12 islets isolated from 4 donors. Glucagon secretion has been normalised to that at 1 mmol/l glucose (1= 2.2±0.4 pg islet^{-1}h$_{-1}$). ***p<0.001 vs 1 mmol/l glucose; 1-way ANOVA with Dunnett's post-hoc test). **(b)** As in (**a**) but testing the effects of GLP-1(9-36) at 1, 6 and 20 mmol/l glucose. Each data point represents a unique group of 12 islets isolated from 4 donors. Glucagon secretion has been normalised to that at 1 mmol/l glucose (1=9.2±1pg islet^{-1} h^{-1}). ***p<0.001 vs 1 mmol/l glucose. †p<0.05 vs 6 mmol/l glucose alone (RM one-way ANOVA with the Geisser-Greenhouse correction of the mean values for each group of the individual experiments).

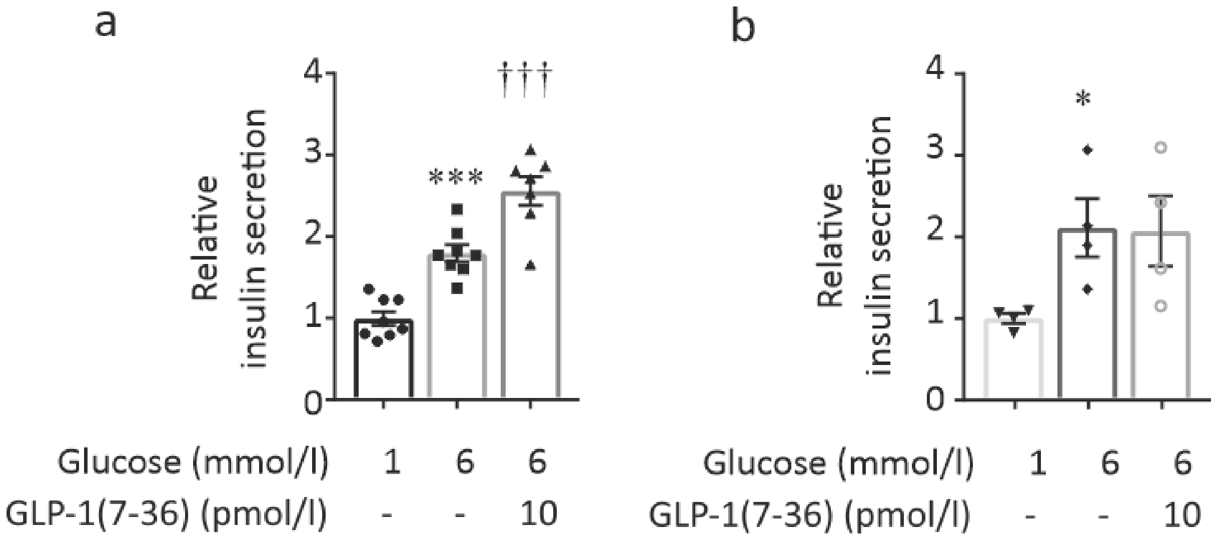

ESM Fig. 2 GLP-1's insulinotropic effect requires GLP1-R. **(a-b)** Effects of 10 pmol/l GLP-1(7-36) on insulin secretion. Glucose was included in the medium at 1 or 6 mmol/l as indicated. Islets isolated from wild-type (**a**) and *Glp1r*$^{-/-}$ mice (**b**). Each data point represents a unique group of 12 islets isolated from 4 mice of each genotype. Insulin secretion normalised to that at 1 mmol/l glucose. *$p<0.05$, ***p<0.001 vs 1 mmol/l glucose for each genotype; †††$p<0.001$ vs 6 mmol/l glucose in wild-type islets (1-way ANOVA with Dunnett's post-hoc test).

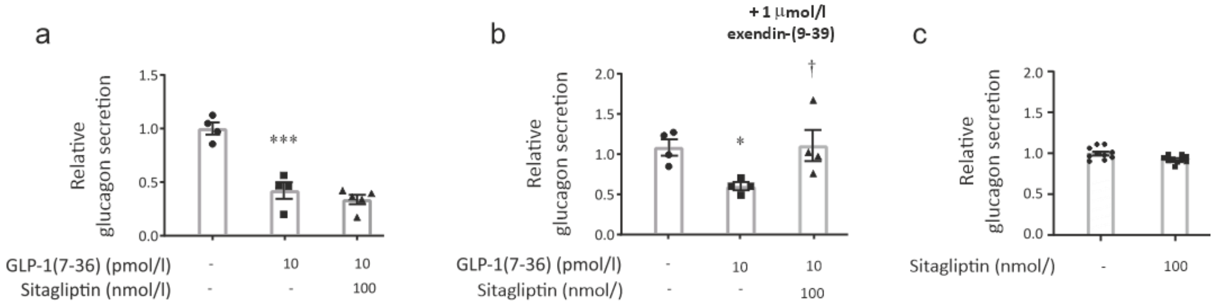

ESM Fig. 3 GLP-1(7-36)'s GLP-1R-independent effect requires degradation. **(a-b)** Effects of 10 pmol/l GLP-1(7-36) on glucagon secretion in the presence of sitagliptin (100 nmol/l) in the absence (a) or presence (b) of exendin-(9-39). Each data point represents a unique group of 12 islets isolated from 4 mice. Glucagon secretion has been normalised to that at 1 mmol/l glucose (1= 4.1±0.2 pg islet^{-1} h^{-1} (**a**) and 2.3±0.26 pg islet^{-1} h^{-1} (**b**)). *p<0.05, **p<0.01, ***p<0.001 vs no GLP-1(7-36); †p<0.05 vs 10 pmol/l GLP-1(7-36) vs no sitagliptin (1-way ANOVA with Dunnett's post-hoc test). **(c)** Effect of sitagliptin (100 nmol/l) at 1 mmol/l glucose alone. Each data point represents a unique group of 12 islets isolated from 8 mice. Glucagon secretion has been normalised to that at 1 mmol/l glucose (1= 4.07±0.2 pg islet^{-1} h^{-1}).

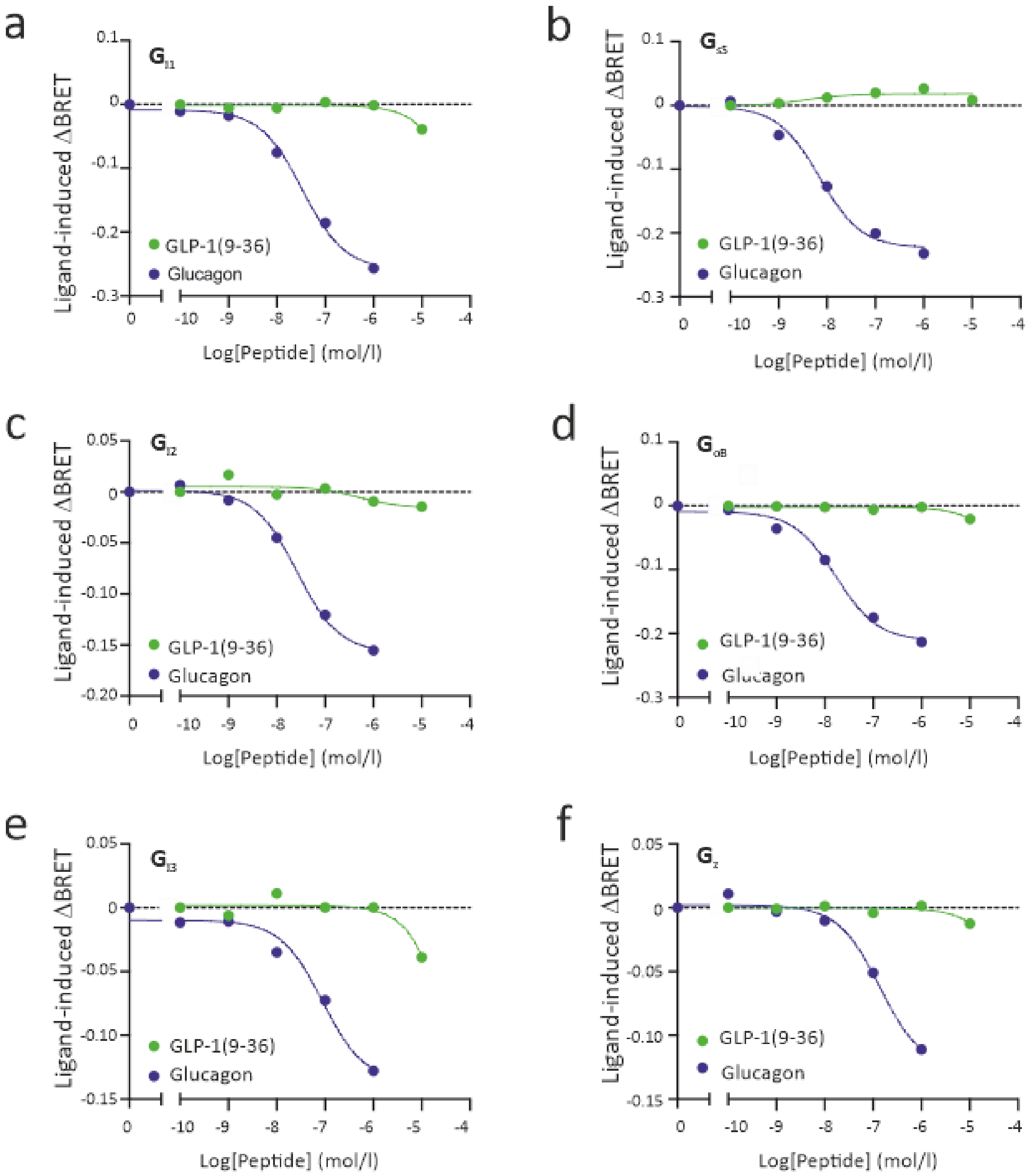

ESM ESM Fig. 4 GCGR activation by glucagon and GLP-1 (9-36). **(a-f)** Effects of increasing concentrations of glucagon and GLP-1(9-36) on dissociation of the different GTP-binding protein alpha-subunits (indicated to the right of the vertical axes in panels a-f) from GCGR expressed in HEK293T cells using the TRUPATH biosensor platform. Effects are expressed as the ligand-induced change in BRET (ΔBRET) against concentration of glucagon and GLP-1(9-36). Data representative of 3-5 replicates (in duplicate). See also ESM Table 4.

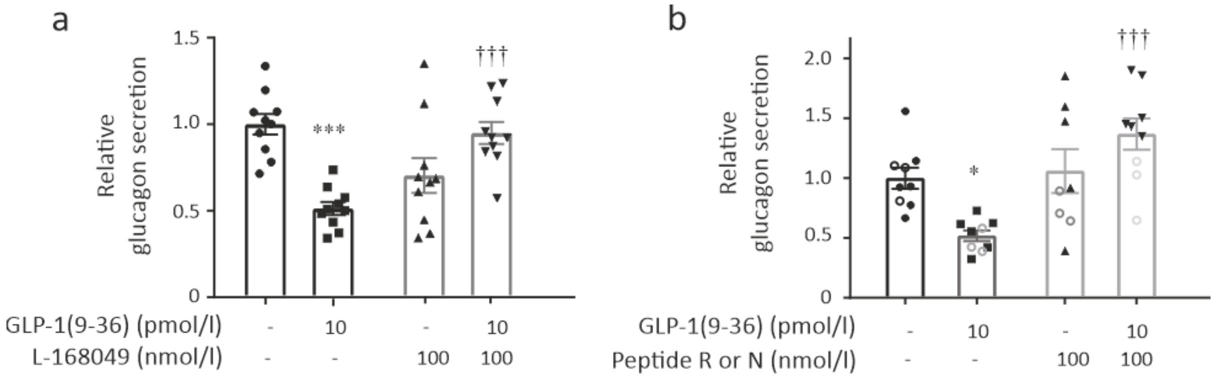

ESM Fig. 5 GRAs prevent glucagonostatic effects of GLP-1(9-36). **(a)** Effects of 10 pmol/l GLP-1(9-36) on glucagon secretion in the absence or presence of 100 nmol/l L-168049. Each data point represents a unique group of 12 islets isolated from 6 mice. Glucagon secretion has been normalised to that at 1 mmol/l glucose (1=5.9±0.3pg islet^{-1}h^{-1}). **p*<0.05 versus no GLP-1(9-36), †††*p*<0.001 vs 10 pmol/l GLP-1 in the absence of L-168049 (one-way ANOVA followed by Dunnet’s post-hoc). Glucagon secretion normalised to rate of release at 1 mmol/l glucose (n=8-9 from 3 donors). **(b)** Glucagon secretion in human islets in the absence and presence of GLP-1(9-36). The GCGR antagonists desHis^{1}Pro^{4}Glu^{9}glucagon (”Peptide N) or desHis^{1}Pro^{4}Glu^{9}Lys^{12}PAL-glucagon (‘Peptide R) were included in the incubation medium at a concentration of 100 nmol/l as indicated. For display, data with the two antagonists have been pooled. Each data point represents a unique group of 12 islets isolated from 3 donors. Glucagon secretion has been normalised to that at 1 mmol/l glucose (1=7.2±0.7 pg islet^{-1}h^{-1}). **p*<0.05 vs no GLP-1(9-36), †††*p*<0.001 vs 1 pmol/l GLP-1(9-36) in the absence of peptide R or N (one-way ANOVA followed by Dunnet’s post-hoc).

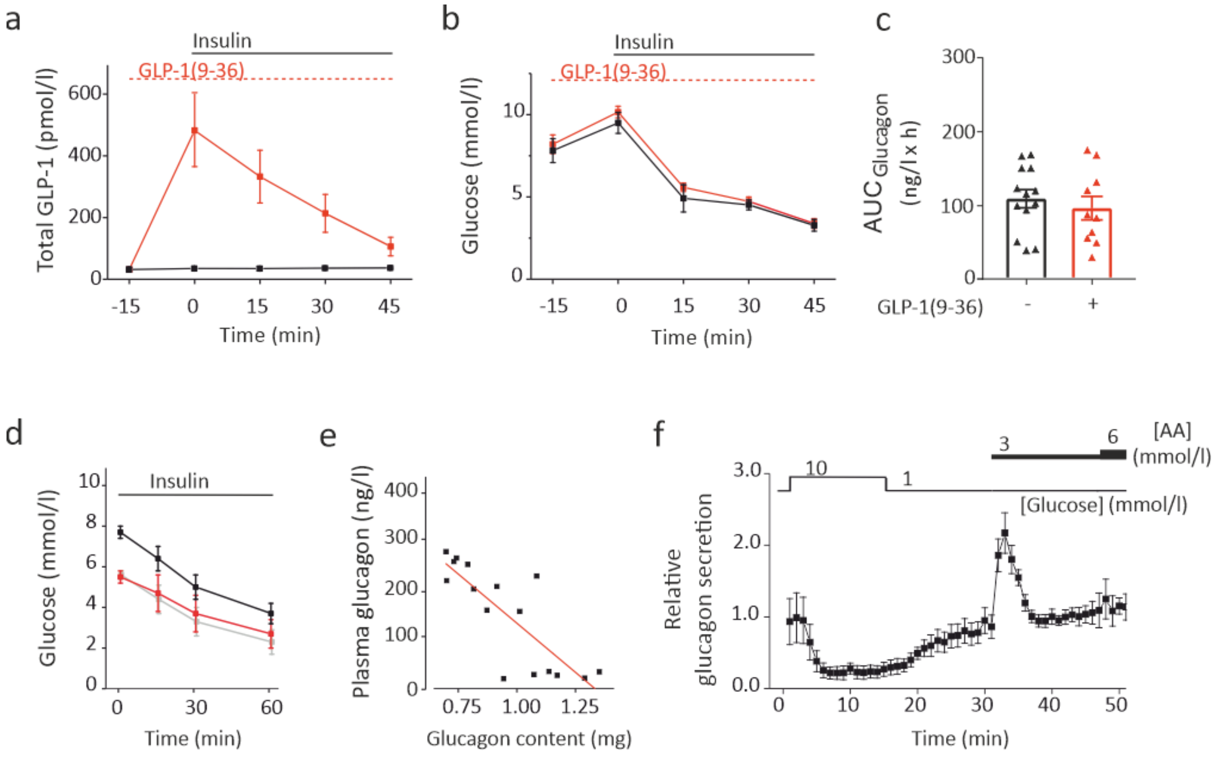

ESM Fig. 6 Effects of GLP-1(9-36) *in vivo*. **(a)** Total plasma GLP-1 measured with (red symbols) or without (black symbols) injection of GLP-1(9-36) (100 µg/kg body weight; at t=-15 min). GLP-1(9-36) was injected intraperitoneally (ip) and samples were taken at indicated times. At t=0 min, insulin (0.75 U/kg was injected ip. Data are mean values ±S.E.M. of 7 mice in each group. **(b)** Plasma glucose measured during insulin-induced hypoglycaemia in (a). Plasma glucose values are not statistically different at any time point (n=13 saline and n=14 GLP-1(9-36)). **(c)** Dot plots of areas under the curve (AUCs) measured during 45min following injection of insulin at t=0min in the absence (black) or presence (red) of exogenous GLP-1(9-36) when GLP-1(9-36) was co-injected with insulin (0.75 U/kg). **(d)** Lowering of plasma glucose following injection of insulin (0.75U/kg body weight) under control conditions (black) and after pretreatment with REMD2.59 in the absence (red) or presence (grey) of GLP-1(9-36) (100 ng/g body weight; injected at t=-15 min; red). **(e)** Relationship between pancreatic glucagon content and plasma glucagon (r=-0.816). **(f)** Glucagon secretion measured in the perfused mouse pancreas at 10 and 1 mmol/l glucose as indicated. Increasing glucose reversibly inhibited glucagon secretion by 80%. A cocktail of amino acids (glutamine, alanine and arginine, equal amounts) was included at a total concentration of 3 and 6mmol/l in the perfusion medium at 1 mM glucose as indicated. Mean values ± S.E.M. of 5 experiments. Secretion rates have been normalised to that at 1 mmol/l glucose (1=86±17 pg/min; n=10 mice). Stimulatory effect of 3 mmol/l AAs is statistically significant (p<0.005 by ANOVA).

ESM Table 1. Human islet donor details

**Parameter**	
Age (years) ([Range]	48 [3, 80]
BMI (kg x m^{-2})	23.8 [15.9, 34]
Number of donors	22
Male/Female	13/9
Healthy/Type 2 diabetes	17/5

Human pancreatic islets were isolated (with ethical approval and clinical consent). Human islets were usually released for experimental work within 24h of islet isolation. During the interval between islet isolation and the hormone secretion studies, islets were maintained in complete RPMI medium containing 5mmol/l glucose for up to 2 days prior to the experiments. Donors with type-2 diabetes were identified based on clinical history and/or measured HbA1C.Data are givem as mean values and the range.

ESM Table 2

**Salts (mmol/l)**	EC1	EC2	EC3
NaCl	120	140	140
KCl	4.7	3.6	3.6
$CaCl_2$	2.5	2.5	2.6
KH_2PO_4	1.2	0.5 (NaH_2PO_4)	1 (NaH_2PO_4)
$MgSO_4$	1.2	0.5	1.2
$NaHCO_3$	25	2	5
HEPES	10 (pH 7.4 with NaOH)	5 (pH 7.4 with NaOH)	10 (pH 7.4 with NaOH)
BSA (%)	0.1 (for secretion)	0.2	0.2
Main application	Mouse islet secretion[1] & TIRF imaging	Human islet secretion	PKA activity measurements

Composition of extracellular media used.

[1]Except for experiments in Fig. 2C and ESM Figs 2-4, 5h and 6-7 in which EC2 was used.

ESM Table 3

**Gα-Rluc8**	Gβ	Gγ-GFP2
sS	3	9
i1	3	9
i2	3	8
i3	3	9
oA	3	8
oB	3	8
Z	3	1

Combinations of TRUPATH constructs used to measure G Protein dissociation.

Combinations are as described in ref. no. [21].

ESM Table 4

	Glucagon			GLP-1(9-36)		
**Gα**	**Emax**	**pEC50 (mol/l)**	**(n)**	**Emax**	**pEC50 (mol/l)**	**(n)**
i1	-0.258±0.012	7.47±0.10	3	-	-	4
i2	-0.157±0.009	7.58±0.14	3	-0.016±0.012	6.30±1.15	3
i3	-0.137±0.014	7.05±0.20	3	-	-	3
oA	-0.210±0.009	7.83±0.10	4	-0.021±0.003*	7.55±-0.52	3
oB	-0.211±0.015	7.78±0.18	5	-	-	4
αz	-0.126±0.017	6.86±0.22	3	-	-	5
sS	-0.223±0.014	8.16±0.16	4	-	-	3

Maximum emission change (Emax: ΔBRET) and concentration (log_{10}) of glucagon and GLP-1(9-36) at which ΔBRET is half-maximal (pEC50) derived from the experiments in ESM Fig. 5. Data are shown for GCGR for indicated Gα subunits. Data are mean values ± S.E.M. of indicated number of experiments (n). -, insufficient data. *p=0.0016 vs baseline; Gαi2 not statistically significant (p=0.067). Effects of glucagon statistically significant for all Gα.

Checklist for reporting human islet preparations used in research

Adapted from Hart NJ, Powers AC (2018) Progress, challenges, and suggestions for using human islets to understand islet biology and human diabetes. Diabetologia https://doi.org/10.1007/s00125-018-4772-2

**Islet preparation**	**1**	**2**	**3**	**4**	**5**	**6**	**7**	**8[a]**
**MANDATORY INFORMATION**								
Unique identifier	HP10-39	HP10-28	HP10-29	HP10-44	HP11-44	HP11-37	HP14-28	
Donor age (years)	38	47	24	28	24	59	50	
Donor sex (M/F)	M	M	M	F	M	F	F	
Donor BMI (kg/m^2)	34	25.7	25	23	25	23	26	
Donor HbA_{1c} or other measure of blood glucose control	-							
Origin/source of islets[b]	Oxford	Oxford	Oxford	Oxford	Oxford	Oxford	Oxford	
Islet isolation centre	Oxford	Oxford	Oxford	Oxford	Oxford	Oxford	Oxford	
Donor history of diabetes? Please select yes/no from drop down list	No	No	No	No	No	No	No	
Diabetes duration (years)								
Glucose-lowering therapy at time of death[c]								
Donor cause of death	ICH	ICH	RTA	DCD	ICH	ICH	ICH	
Warm ischaemia time (h)				0.2	0.5			
Cold ischaemia time (h)	5	7.5	7	9	3.5	10	5	

Estimated purity (%)	70	80	70			70	50	
Estimated viability (%)	90					80	79	
Total culture time (h)[d]								
Glucose-stimulated insulin secretion or other functional measurement[e]	2- to 4-fold	2-fold	7-fold	2-fold	2-fold	3-fold	2-fold	
Handpicked to purity? Please select yes/no from drop down list	Yes	Yes	Yes	Yes	Yes	Yes	Yes	
Additional notes								

[a]If you have used more than eight islet preparations, please complete additional forms as necessary
[b]For example, IIDP, ECIT, Alberta IsletCore
[c]Please specify the therapy/therapies
[d]Time of islet culture at the isolation centre, during shipment and at the receiving laboratory
[e]Please specify the test and the results

Checklist for reporting human islet preparations used in research

Adapted from Hart NJ, Powers AC (2018) Progress, challenges, and suggestions for using human islets to understand islet biology and human diabetes. Diabetologia https://doi.org/10.1007/s00125-018-4772-2

Islet preparation	1	2	3	4	5	6	7	8[a]
**MANDATORY INFORMATION**								
Unique identifier	H2307	H2109	R249	H2510	H2516	R390	R392	HP14-29
Donor age (years)	58	65	62	52	55	3	8	44
Donor sex (M/F)	F	F	F	M	M	M	F	F
Donor BMI (kg/m^2)	26.7	20.8	22.2	26.9	22.4	19.8	15.9	29
Donor HbA_{1c} or other measure of blood glucose control<br><br>mmol/mol (%)	41 (5.9%)	41 (5.9%)	-	17 (3.7%)	16 (3.6%)	10 (3.1%)	29 (4.8%)	36 (5.4%)
Origin/source of islets[b]	Uppsala/Nordic Network for clinical islet transplantation	Uppsala/Nordic Network for clinical islet transplantation	Alberta	Uppsala/Nordic Network for clinical islet transplantation	Uppsala/Nordic Network for clinical islet transplantation	Alberta	Alberta	Oxford
Islet isolation centre	Nordic Network for clinical islet transplantation	Uppsala/Nordic Network for clinical islet transplantation	Alberta Isletcore, Edmonton	Nordic Network for clinical islet transplantation	Nordic Network for clinical islet transplantation	Alberta Isletcore, Edmonton	Alberta Isletcore, Edmonton	Oxford
Donor history of diabetes? Please select yes/no from drop down list	No	No	No	No	No	No	No	No
**If Yes, complete the next two lines if this information is available**								
Diabetes duration (years)								
Glucose-lowering therapy at time of death[c]								

RECOMMENDED INFORMATION								
Donor cause of death	DBD - Myocardial infarction	Trauma	DCD - Cardio-Circulatory			NDD - Neurological	NDD - Neurological	ICH
Warm ischaemia time (h)	3.5	2.5		2.5	3			0.9
Cold ischaemia time (h)	19	6.5	19	12	9	16.5	12.5	4
Estimated purity (%)			40%			75%	80%	50%
Estimated viability (%)								
Total culture time (h)[d]								
Glucose-stimulated insulin secretion or other functional measurement[e]	TIRF imaging	TIRF imaging	9.2-fold to 10G TIRF imaging	4-fold	5-fold	4-fold	3.5-fold	3-fold
Handpicked to purity? Please select yes/no from drop down list	Yes	Yes	Yes	Yes	Yes	Yes	Yes	Yes
Additional notes								

[a]If you have used more than eight islet preparations, please complete additional forms as necessary
[b]For example, IIDP, ECIT, Alberta IsletCore
[c]Please specify the therapy/therapies
[d]Time of islet culture at the isolation centre, during shipment and at the receiving laboratory
[e]Please specify the test and the results

Checklist for reporting human islet preparations used in research

Adapted from Hart NJ, Powers AC (2018) Progress, challenges, and suggestions for using human islets to understand islet biology and human diabetes. Diabetologia https://doi.org/10.1007/s00125-018-4772-2

Islet preparation	1	2	3	4	5	6	7	8[a]
MANDATORY INFORMATION								
Unique identifier	H2610	H2613	H2203	H2238	H2611	H2618	R472	
Donor age (years)	62	65	59	73	51	80	49	
Donor sex (M/F)	M	M	M	F	M	M	M	
Donor BMI (kg/m^2)	22	22	20.9	22.3	NA	27.7	20.4	
Donor HbA_{1c} or other measure of blood glucose control	36 (5.4%)	38 (5.6%)	45 (6.3%)	44 (6.2%)	58 (7.5%)	45 (6.3%)	49 (6.6%)	
Origin/source of islets[b]	Uppsala/Nordic Network for clinical islet transplantation	Uppsala/Nordic Network for clinical islet transplantation	Uppsala/Nordic Network for clinical islet transplantation	Uppsala/Nordic Network for clinical islet transplantation	Uppsala/Nordic Network for clinical islet transplantation	Uppsala/Nordic Network for clinical islet transplantation	Alberta	
Islet isolation centre	Nordic Network for clinical islet transplantation	Uppsala/Nordic Network for clinical islet transplantation	Nordic Network for clinical islet transplantation	Uppsala/Nordic Network for clinical islet transplantation	Nordic Network for clinical islet transplantation	Uppsala/Nordic Network for clinical islet transplantation	Alberta Isletcore, Edmonton	
Donor history of diabetes? Please select yes/no from drop down list	No	No	Yes	Yes	Yes	Yes	Yes	
**If Yes, complete the next two lines if this information is available**								
Diabetes duration (years)							3 years	
Glucose-lowering therapy at time of death[c]					Metformin			

RECOMMENDED INFORMATION								
Donor cause of death			Cardiac arrest	SAH			NDD - Neurological	
Warm ischaemia time (h)	2.5	2.5	3	2.5	2.5	2		
Cold ischaemia time (h)	19.5	8	2.5	17.5	13	7	11	
Estimated purity (%)							75	
Estimated viability (%)								
Total culture time (h)[d]								
Glucose-stimulated insulin secretion or other functional measurement[e]	SI 3.6		TIRF imaging	TIRF imaging		SI 11.1	2.3-fold to 10G	
Handpicked to purity? Please select yes/no from drop down list	Yes	Yes	Yes	Yes	Yes	Yes	Yes	
Additional notes								

[a]If you have used more than eight islet preparations, please complete additional forms as necessary
[b]For example, IIDP, ECIT, Alberta IsletCore
[c]Please specify the therapy/therapies
[d]Time of islet culture at the isolation centre, during shipment and at the receiving laboratory
[e]Please specify the test and the results
